# Spider mite egg extract modifies Arabidopsis response to future infestations

**DOI:** 10.1038/s41598-021-97245-z

**Published:** 2021-09-06

**Authors:** Dairon Ojeda-Martinez, Manuel Martinez, Isabel Diaz, M. Estrella Santamaria

**Affiliations:** 1 Centro de Biotecnología y Genómica de Plantas, Universidad Politécnica de Madrid – Instituto Nacional de Investigación y Tecnología Agraria y Alimentaria, Madrid, Spain; 2 Departamento de Biotecnología-Biología Vegetal, Escuela Técnica Superior de Ingeniería Agronómica, Alimentaria y de Biosistemas, Universidad Politécnica de Madrid, Madrid, Spain

**Keywords:** Biotechnology, Plant sciences

## Abstract

Transcriptional plant responses are an important aspect of herbivore oviposition studies. However, most of our current knowledge is derived from studies using Lepidopteran models, where egg-laying and feeding are separate events in time. Little is known regarding plant response to pests where females feed and oviposit simultaneously. The present study characterized oviposition-induced transcriptomic response of Arabidopsis to *Tetranychus urticae* egg extracts. Transcriptional evidence indicates that early events in plant response to the egg extract involve responses typical to biotic stresses, which include the alteration in the levels of Ca^2+^ and ROS, the modification of pathways regulated by the phytohormones jasmonic acid and ethylene, and the production of volatiles and glucosinolates as defence mechanisms. These molecular changes affect female fertility, which was significantly reduced when mites fed on plants pre-exposed to the egg extract. However, longer periods of plant exposure to egg extract cause changes in the transcriptional response of the plant reveal a trend to a decrease in the activation of the defensive response. This alteration correlated with a shift at 72 h of exposition in the effect of the mite feeding. At that point, plants become more susceptible and suffer higher damage when challenged by the mite.

## Introduction

Research on herbivore oviposition has recently become a topic of great interest, where numerous results are emerging. Out of the wide array of known plant defences in response to eggs, such as ovicidal compound production, local tissue necrosis, and egg crushing^1–3^, information on the complex network of transcriptional plant responses is of specific interest. Such studies have described early Ca^2+^, electrical and Reactive Oxygen Species (ROS) imbalances^3,4^ and have revealed the importance of Jasmonic Acid (JA) and Salicylic Acid (SA) as the major plant hormonal regulators in response to oviposition^5^. In Arabidopsis, responses associated with phytophagous insect eggs are mainly dominated by SA defence cascades^6,7^, and are similar to those activated by pathogens^6,8–10^. Most of the aforementioned studies have been performed using lepidopteran species, which facilitate experimental procedures due to lepidopteran egg size. In addition, the spatial and temporal separation of lepidopteran oviposition and feeding events simplifies the analysis of plant transcriptional responses. However, the lepidopteran models do not represent all plant–herbivore interaction events of this nature, considering that many pests oviposit and feed at the same time. Arthropod species following this pattern belong to diverse and vast groups including hemipterans, coleopterans, and mites^11–15^. A transcriptional study exploring such a model has been performed previously^11^. In that study, genes altered by the oviposition of a beetle and associated biological processes were studied. However, much remains to be answered for pests that conform to this pattern of eating-oviposition. Consequently, to better comprehend the effect of simultaneous pest oviposition and feeding in plants, it becomes crucial to delve into the molecular mechanisms of plants triggered by more model species of different classes.

*Tetranychus urticae* Koch (Acari: Tetranychidae) is a spider mite known for its voracity as a pest, feeding on 1,100 plant species, being 150 of them of agronomic importance^16^. Added to its cosmopolitan characteristics, a rich genetic repertoire that helps it to resist pesticides makes this herbivore a formidable foe in agricultural production^17^. *T. urticae* is easily reared in laboratory conditions, its genome has been sequenced and has the ability to feed on model species such as *Arabidopsis thaliana*, all of which makes it a good mite model for herbivory studies^17,18^. During the early stages of colonization, adult females settle and oviposit on the plant surface^19^ and feed by piercing between epidermal cells or through the stomata, accessing the parenchymal tissue^20^. At later stages of colonization, females protect their eggs by laying them on densely intertwined silk^21^. Spider mite eggs have a spherical shape and a diameter size of about 150 µm^22^, which varies depending on the offspring sex, female eggs being larger than male eggs^23^.

To understand feeding-induced responses, a transcriptional investigation on the *A. thaliana*-*T. urticae* model has been previously analysed^15,24^. This analysis revealed a strong induction of defence-related genes, secondary metabolite production, and JA, SA, and ethylene (ET) cascades. However, the physiology and behaviour of *T. urticae* makes it extremely difficult to separate feeding from other simultaneous events such as oviposition, defecation, and mechanical stimulation of the plant. Thus, the previously obtained transcriptional results are the consequence of a merge of signals caused by the spider mite. In contrast, eggs may be isolated and used to determine the plant transcriptional response exclusively due to mite oviposition. In this context, diverse plant responses have been reported^2,25^. Either eggs induce plant defences that directly kill them or act as warning signals to trigger plant responses leading to impaired herbivore performance or to attract natural enemies of the pest. All these responses are determined by specific molecules present in the egg, also named egg-associated molecular patterns (EAMP), which have been scarcely studied. Whether plant responses are elicited by EAMPs located inside or on the outer surface of the eggs is a matter of discussion. In *Pieris brassicae*, eggs are covered by secretions released from the female accessory reproductive glandular reservoir, which are able to elicit a plant defence response^26,27^. Likewise, treatment of *A. thaliana* leaves with the supernatant or lipid fractions of crushed eggs of *P. brassicae* mimics natural egg deposition^5,8^. Recently, Stahl et al.^28^ showed that purified phosphatidylcholines from *P. brassicae* eggs trigger similar defence responses in Arabidopsis than egg extracts. These compounds diffuse from inside the eggs to the leaves at concentrations that are sufficient to induce plant responses.

Based on these findings, we focused our study in the capacity of *T. urticae* eggs to trigger a plant response in Arabidopsis. To maximize the effects of potential elicitors from the egg, an extract preparation was used in performing experiments*.* To our knowledge, this is the first study investigating the effect of egg extract from an herbivorous chelicerate on plant transcriptional dynamics. Here, we present transcriptional evidence indicating that the spider mite egg extract alters molecular and electrical signals such as Ca^2+^ and ROS balance, and induces upregulation of JA and ET cascades. Additionally, phenotyping assays revealed a significant reduction in female fertility upon feeding on egg extract pre-treated plants, while feeding increased on plants pre-treated for 72 h.

## Results

### Egg morphology and histochemical staining

**Figure 1:**
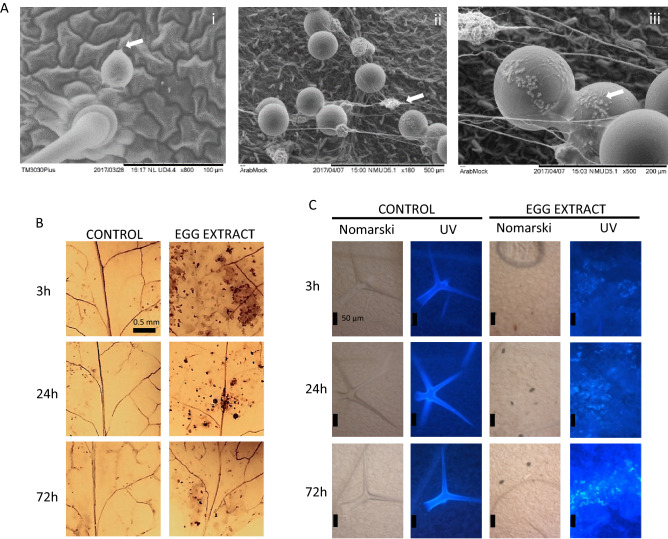
Egg morphology and plant damage upon egg extract deposition. Spider mite egg images were analysed under scanning electron microscopy (**A**). Arrows indicate silk (i, ii) and oviposition fluids (ii, iii). Leaves of three week old Col-0 plants were pre-exposed to 2 µl of the spider mite egg extract for 3, 24 and 72 h, as indicated in each panel. Afterwards, leaves were stained using 3,3-diaminobenzidine (DAB) to identify H_2_O_2_ (**B**) and aniline blue to evaluate callose deposition (**C**). Nomarski images are also shown. Scale bars are indicated in the figures. UV images in non-treated plants show the fluorescence of trichomes as a technical positive control.

As previously described by Tuan et al.^21^, spherical spider mite eggs of approximately 150 µm in diameter are observed to be laid on leaves or suspended on silk (Fig. 1A). Fluid content was observed mainly joining the eggs together with multiple undetermined depositions on the egg surface, alongside the usual dense silk fibres (Fig. 1A).

To explore the possible effects of the spider mite egg extract on defence responses, peroxide accumulation, cell death, and callose deposition were characterized. The accumulation of H_2_O_2_ at the leaf deposition site of the egg extract was detected by the brown colour of oxidized 3,3-diaminobenzidine tetrachloride hydrate (DAB) used as substrate in the histochemical assays. The highest content of H_2_O_2_ was observed at 3 hpt (hours post-treatment) (Fig. 1B), fading with time. Callose deposition was visualized by the fluorescence emitted at the egg extract deposition site after blue aniline staining. Fluorescence was detected at 3 hpt, seemingly present only on epidermal tissue (Fig. 1C). At later points, the fluorescence was extended to mesophyll cells, being observed the highest response at 72 hpt. In contrast, no obvious cell death processes were found when the treated leaves were analysed using trypan blue (TB) staining (data not shown).

### Transcriptional response to spider mite egg extract

**Figure 2:**
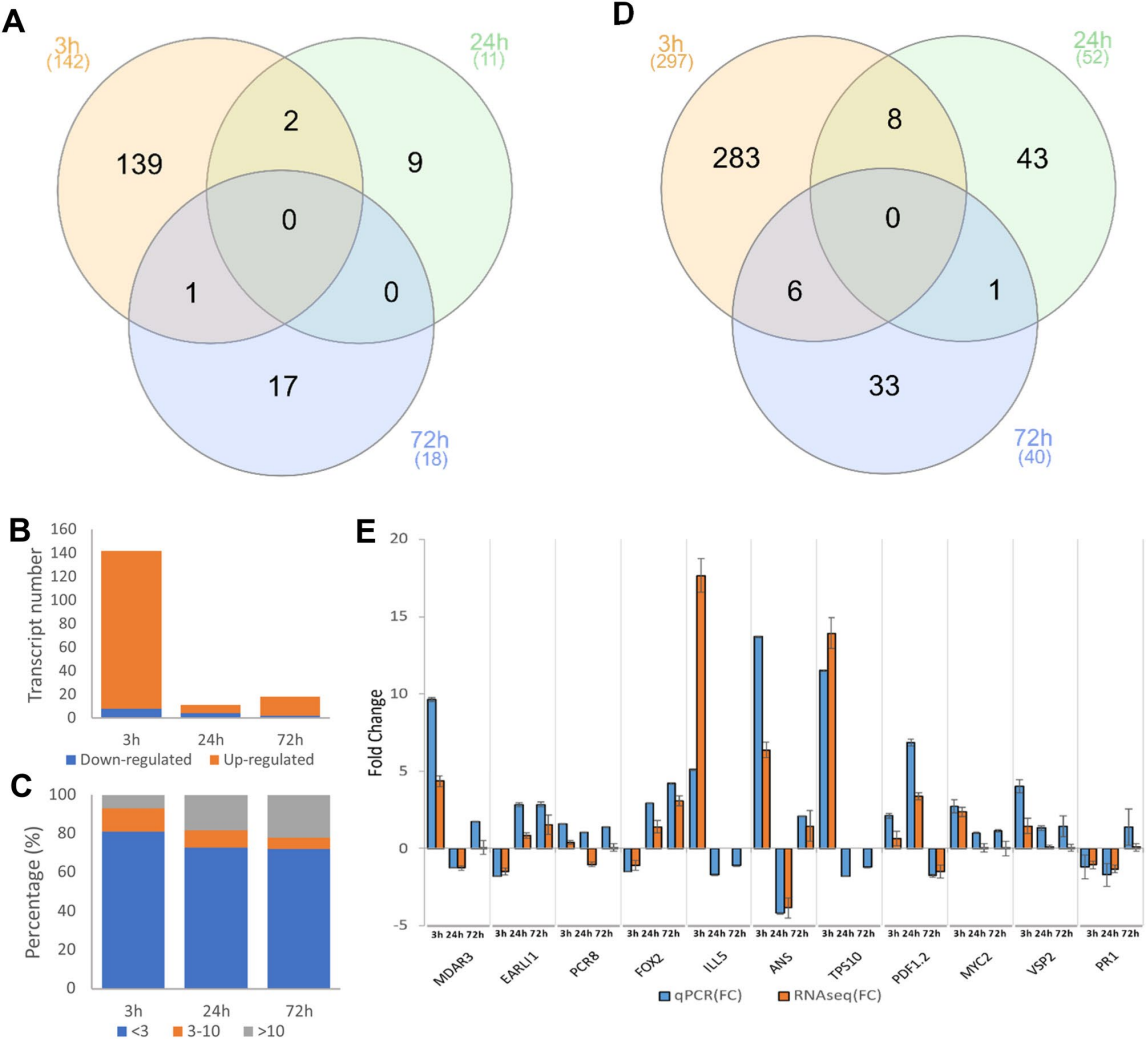
Transcriptional response of *Arabidopsis* rosettes to spider mite egg extract. (**A**) Venn diagram of the DETs identified at each time point (3, 24 and 72 hpt) under analysis (**B**) A comparison among the total amount of DETs up-regulated and down-regulated at each time point. (**C**) Proportion of the transcripts whose FC modulus was between the ranges 1.4 ≤ FC ≤ 3 (in blue), 3 < FC ≤ 10 (in orange) and FC > 10 (in grey) are depicted. (**D**) Venn diagram of the DEGs obtained from the Lancaster aggregation method performed on the full list of transcripts. (**E**) RT-qPCR validation of the RNA-seq gene expression using eleven genes; both data sets significantly correlate (r_s_ = 0.96, p < 0.001). qPCR error bars represent the range of the fold changes calculated as 2^−(ΔΔCt)^ and RNA-seq error bars as log_2_(FCs), (n = 3). The represented DETs were those whose log_2_(FC) ≥ 0.5 and p_adj_ < 0.05 (n = 3), and the DEGs, those whose p_adj_ < 0.05.

To analyse the transcriptional response of the plant to the egg extract, samples were collected from rosettes upon 3, 24, and 72 h of treatment. Three replicates were collected per treatment and control for each time point and subjected to RNA-seq. Approximately 500 million paired-end reads were obtained, having an average of 27 million reads per sample (Table S1). Over 98% of the aforementioned reads were mapped to the *A. thaliana* reference genome (TAIR10; https://www.arabidopsis.org) and over 96% were uniquely mapped (Table S2). Mapped regions for all samples were mostly exonic (over 98%), whereas intergenic and intronic regions accounted for 0.55% of the reads mapped. Transcriptomic data processing based on a Kallisto-Sleuth pipeline produced a list of differentially expressed transcripts (DETs). Transcript-level *p*-values were aggregated by means of the Lancaster method, generating an accurate and sensitive list of differentially expressed genes (DEGs). Working together with both DEGs and DETs during the present study allowed the analysis of individual transcript dynamics, as well as the identification of genes even under cancellation, domination, or collapsing processes. RNA-seq analysis revealed a total of 142, 11, and 18 DETs that were identified at 3, 24, and 72 hpt, respectively (Supplemental File 1). Multiple DETs corresponding to the same gene were identified at 3 and 72 hpt; while at 24 hpt every gene had a single transcript regulated. No DET was identified as differentially regulated at all time points (Fig. 2A). The two DETs differentially expressed at 3 and 24 hpt were AT2G24850.1 and AT3G16400.1. Both of them were detected as up-regulated at 3 hpt and down-regulated at 24 hpt. A single transcript was upregulated at both 3 and 72 hpt: AT4G17500.1. The highest upregulation of DETs was detected at 3 hpt (Fig. 2B). At 24 hpt, the processes of downregulation were proportionally the highest among the three time points, accounting for 36% of the total DETs regulated (Fig. 2B). Fold change behaviour for the identified DETs was similar among the three time points (Fig. 2C). Low FC responses were the majority (over 70% for all time points), whereas high FC profiles were rarer (Fig. 2C). Subsequently, a list of DEGs was extracted from the DETs (Supplemental File 2). The transcriptional response involved a considerable number of genes, 297, at the early exposition to the egg extract (Fig. 2D). At 24 and 72 hpt, the response involved the regulation of fewer genes than at 3 hpt, 52 and 40, respectively (Fig. 2D). All genes corresponding to the DETs were present in the lists of DEGs except for 6, 2, and 5 genes at 3, 24, and 72 hpt, respectively. To validate the results obtained in the RNA-seq, 11 genes were selected for validation. The expression profile of the selected genes was confirmed by RT-qPCR for each of the time points. The results of both approaches were significantly correlated (Fig. 2E), having a Spearman´s correlation coefficient of r_s_ = 0.963 and p < 0.001.

### Functional analysis

**Figure 3:**
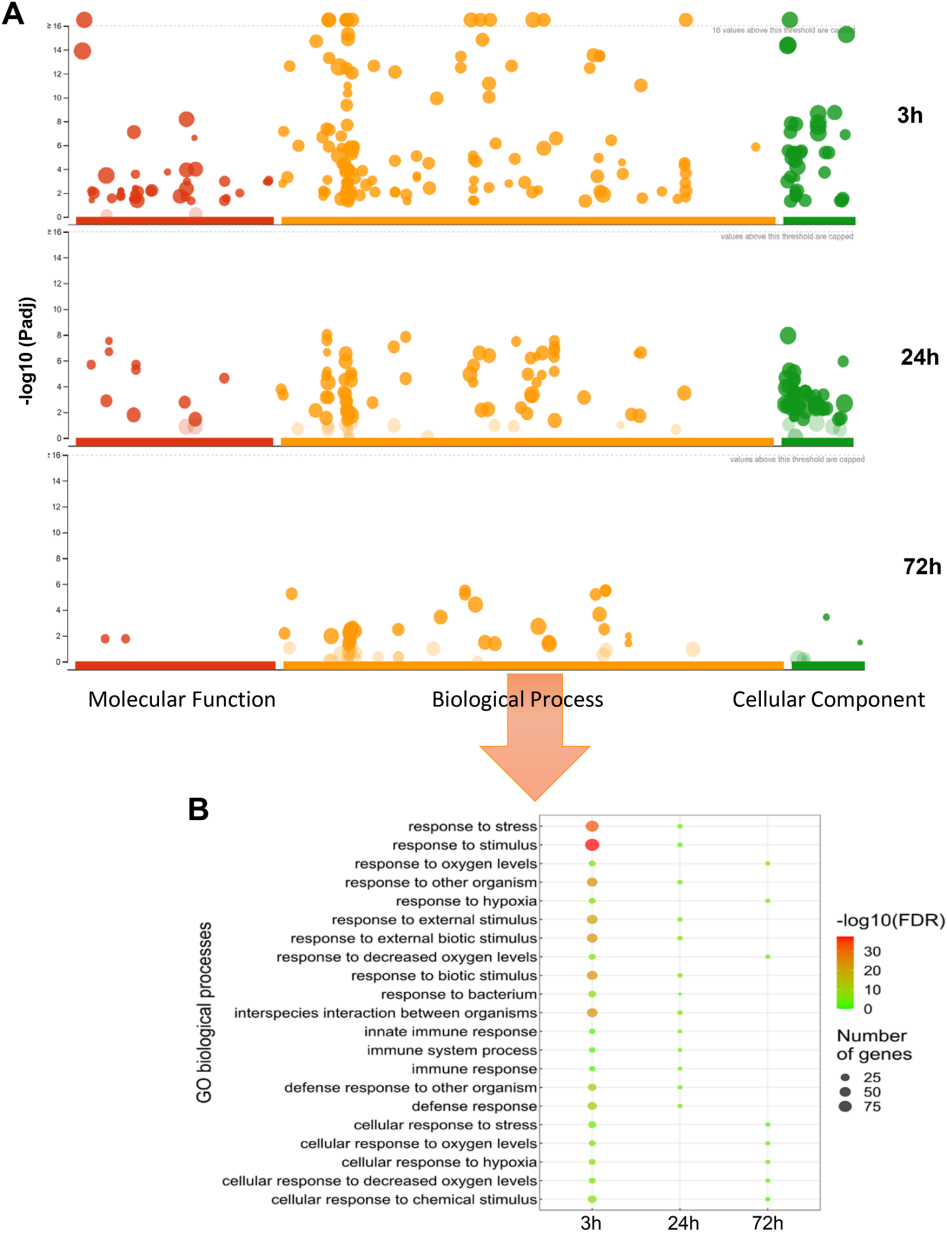
Gene Ontology analysis of the transcriptomic response to the egg extract. (**A**) Manhattan plot of the GO enrichment analysis of the list of DEGs. For all the time points, graphics in orange colour depict Biological Processes, in green Cellular Components and in red Molecular Functions. A threshold was set on each graph to identify the number of ontologies whose –log_10_(p-val.) was ≥ 16. (**B**) Representation of the common 21 BPs enriched upon mite egg extract treatment. The lists of DEGs identified for each time point were classified into enriched BPs. The colour scale goes from the most significant p values (red) to the less significant (green). Dot size indicates number of genes corresponding to each category and time point. Enrichment process was based on a Benjamini–Hochberg test (FDR < 0.05).

To determine whether the DEGs and DETs identified had significantly enriched Molecular Functions (MF), Cellular Components (CC), or Biological Processes (BP), the lists were mapped to Gene Ontology (GO) terms. As a first approach, the g:GOSt tool was used on the lists of DEGs due to their higher number of members. A total of 135, 61, and 28 BP were identified at 3, 24, and 72 hpt (Fig. 3A). The 21 BPs overrepresented in more than one time point are depicted in Fig. 3B (For a more complete list see Fig. S1). After 3 h of exposure to the egg extract, the most represented ontologies were associated with responses to chemical, mechanical, and biotic stresses. Some noticeable defence responses were related to JA, response to biotic stresses such as bacteria or fungi, and responses to ROS (Fig. S1). After 24 h of treatment, the plant response was associated mainly to biotic stimuli, whereas at 72 hpt the response was mostly related to abiotic cues (Fig. 3B and Fig. S1). A total of 39 different MFs were identified at 3 hpt, 10 at 24 hpt, and two at 72 hpt (Fig. 3A and Fig. S2A). A significant overrepresentation of the CCs occurred at 3 hpt, agreeing with the highest amount and variability of the MFs and BPs involved (Fig. 3A). At 24 hpt the overrepresentation was also elevated (41 CCs) and at 72 hpt only two CC ontologies were overrepresented (Fig. 3A and Fig. S2B). Ontologies identified for the DETs are depicted in Figs. S3-S5. Overall, significantly regulated BPs, MFs, and CCs were similar to those observed for DEGs, although with lower numbers of regulated ontologies at any time point.

GO results are inherently redundant due to the fact that the same gene can take part in multiple pathways. Therefore, gathering pathways into a single meaningful process simplifies and enhances the interpretation of the results. To do so and qualitatively compare the results, a network of the identified GOs was produced based on the BP results. To further help the interpretation, similar GO terms were clustered and annotated according to their content. The network constructed from the BPs identified using the list of DEGs is depicted in Fig. S6A. The cluster patterns suggested a more similar response between the samples taken at 3 and 24 hpt (Fig. S6B). Both sample sets presented responses to biotic stresses and some abiotic response-related ontologies, as well as involvement in ion homeostasis. Unlike the first two, the BPs from the last time point were somehow dissimilar. Its processes were less diverse and abundant compared to earlier times and were restricted to abiotic responses or catabolic-mediated regulation processes. Network and clusters constructed from the list of DETs widely agree with those obtained from DEGs (Fig. S7).

### Timeline transcriptomic responses

**Figure 4:**
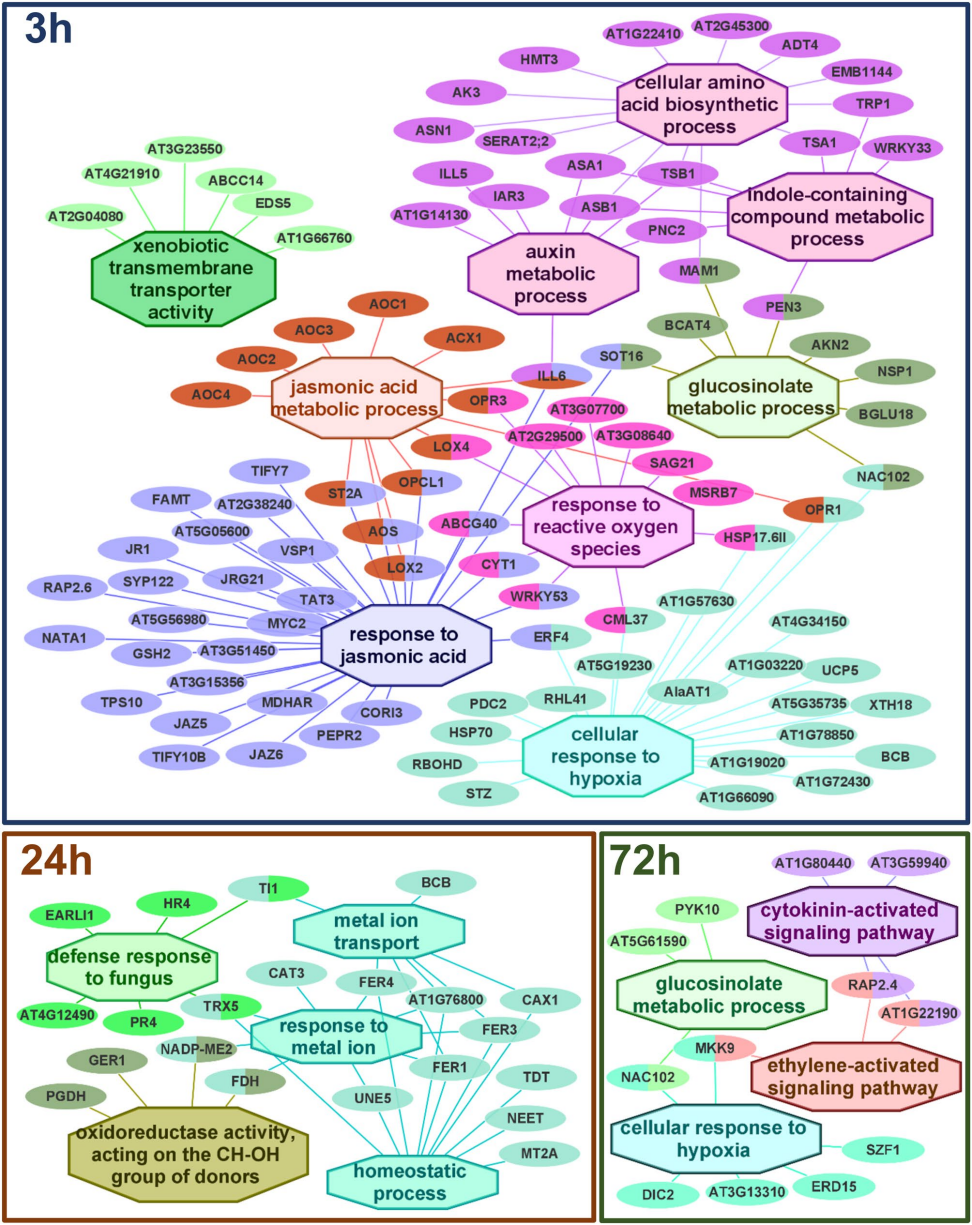
Networks connecting DEGs with enriched biological processes. The BPs enriched from the lists of the DEGs at each time point were used to construct the networks. GO terms are shown as octagons and genes as ellipses. Colours correspond to groups of connected related terms. Enrichment was processed based on a Benjamini–Hochberg test (FDR < 0.05).

To have a deeper insight into the response of *A. thaliana* to egg extract of *T. urticae*, gene enrichment analyses were performed using the ClueGO tool in Cytoscape. This tool permits a more restrictive search, the fusion of related GO terms, and the visualization of genes and GO categories in a unique network. Enriched BPs for the DEGs were identified and networks showing the relationships between BPs and DEGs are depicted in Fig. 4. Additionally, KEGG and PlantCyc tools were explored to highlight enzymatic reactions and relevant pathways.

At 3 hpt, BPs related to the defence response to biotic stimuli were identified. These BPs include the response to reactive oxygen species, the metabolism and response to the JA hormone, and the complex pathway connecting the metabolism of indole-containing amino acids, glucosinolate compounds and auxins. Also, two categories related to the movement of ions and compounds across the membrane were enriched: the cellular response to hypoxia and the xenobiotic transmembrane transporter activity.

The BPs identified are reasonably supported by the presence of DEGs with at least one DET with a fold change higher than two. Many of these highly induced transcripts are closely related to the response of the plant to the JA hormone. Regarding JA-metabolism, several upregulated allene oxide cyclases (*AOC1*, *AOC2*, *AOC3*), and a lipoxygenase (*LOX2*) were induced, which encode enzymes involved in the synthesis of JA. Moreover, upregulated genes also included those encoding the hydrolase ILL6 and the sulfotransferase ST2A, implicated in the conversion of active to inactive JA derived compounds. In the response to JA, genes encoding two TFs (MYC2 and JAZ9) and a lectin (LEC) were highly upregulated, as well as some others encoding proteins with enzymatic activity: the tricyclene synthase TPS10, the L-ornithine N5-acetyltransferase NATA1, the monodehydroascorbate reductase MDHAR, the cystine lyase CORI3, the aminotransferase TAT3, the farnesoic acid carboxyl-O-methyltransferase FAMT, and the strictosidine synthase-like SSL7. Additionally, three genes encoding the 2-oxoglutarate-dependent dioxygenases ANS/JAO4, JRG21/JAO3, and JAO2 were overexpressed. In the complex pathway connecting the metabolism of indole-containing amino acids, glucosinolate compounds, and auxins, only two genes encoding IAA-amino acid hydrolases (*IAR3* and *ILL5*) were highly upregulated. In contrast, five genes involved in the response to reactive oxygen species were overexpressed. The protein products of these genes are two chaperons (HSP17.6B and HSP17.6II), an ABC transporter (ABCG40), a calcium-binding protein (CML37), and a methionine sulfoxide reductase (MSRB7). Finally, the cellular response to hypoxia and the xenobiotic transmembrane transporter activity categories included three and one highly induced genes, respectively. These genes are an NLR of the TIR-only class (*At1g57630*), a TF (*ZAT10/STZ*), a transferase (*At1g19020/SDA1*), and a transporter of the MATE efflux family (*At3g23550/DTX18*).

In addition, eight genes with at least a transcript more than three times induced by egg treatment were not included in the GO groups by the ClueGO analysis. These genes encode a salicylate/benzoate carboxyl methyltransferase (BSMT1), a dioxygenase (DIN11), a ribonuclease (RNS1), an agmatine coumaroyltransferase (ACT), an aspartyl protease (At5g19110), a dimethylnonatriene synthase (CYP94B3), and two transporters, a member of the ABC G family (ABCG33/PDR5) and a probable metal-nicotianamine transporter (YSL5). Also, two DEGs have a transcript at least three times repressed, the glucosylceramidase *At5g49900* and the calcium transporting ATPase *ACA8*.

At 24 h, the identified BPs were mainly related to the homeostatic processes occurring in the cell, with an enrichment of the GO terms metal ion transport, response to metal ions, and oxidoreductase activity. A defensive GO term, defence response to fungus, was also identified. Among the four DEGs with at least one DET with a fold change higher than two, only the induced pEARLI1-like lipid transfer protein 2 was associated with an enriched GO term. The upregulated gene for the defensin-like protein PDF1.2A and the downregulated genes for the TAT3 (aminotransferase) and ABCB28 (ABC transporter) proteins were not associated with any enriched GO term.

At 72 h, four enriched BPs were identified, cellular response to hypoxia, glucosinolate metabolic process, and ethylene and cytokinin-activated signalling pathways. Again, only one DEG out of six with at least one DET with a fold change higher than two was associated with an enriched GO term: the induced gene early responsive to dehydration 15 (*ERD15*). The other five genes not associated with any enriched GO term encode the induced flavin-dependent oxidoreductase FOX2, the pectinesterase inhibitor PME7, and the cytochrome P450 CYP71A12, as well as the repressed callose synthase CALS7 and the E3 ubiquitin-protein ligase At5g58410.

### Phenotyping and fertility assays

**Figure 5:**
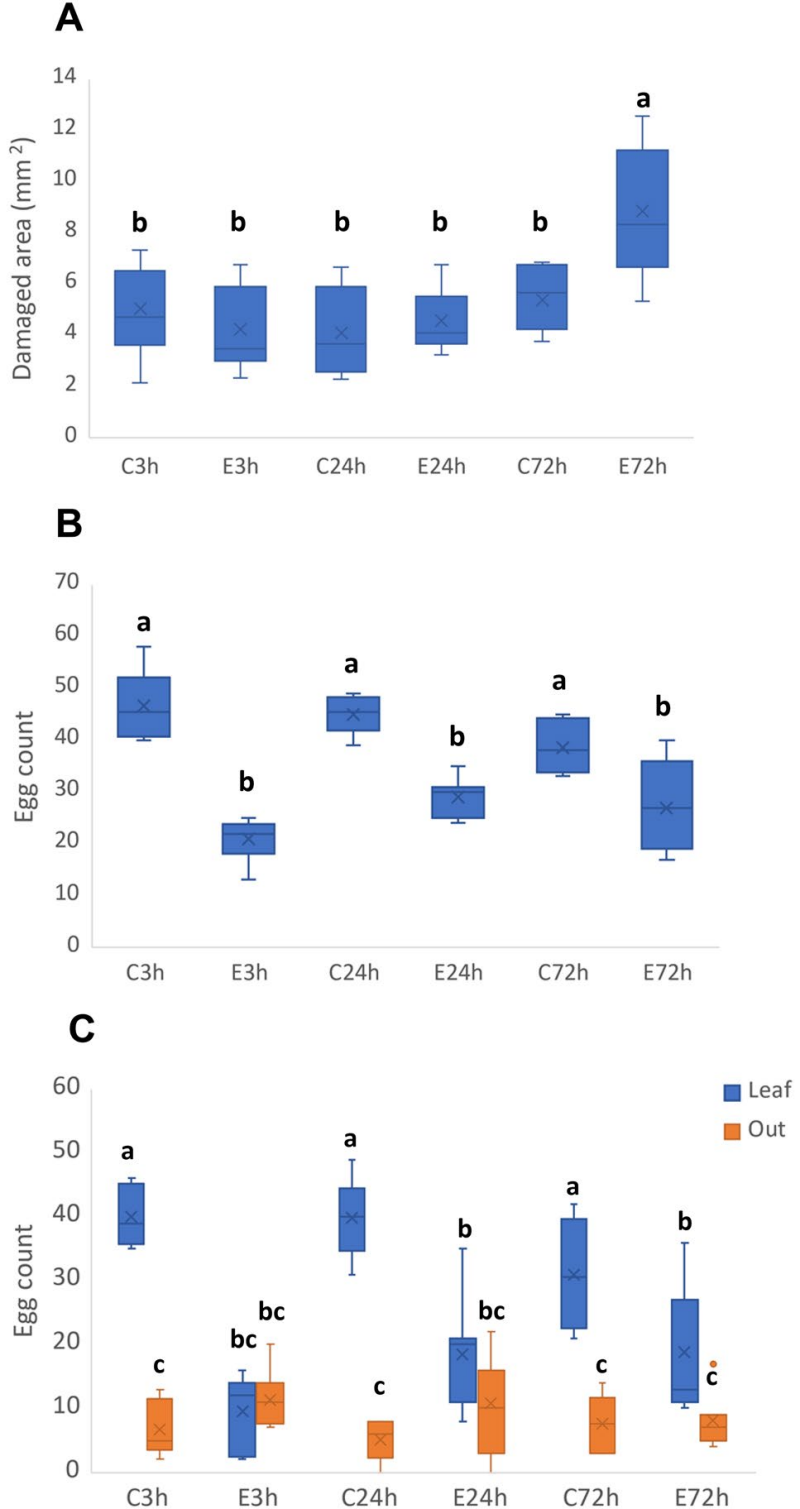
*T. urticae* feeding and fertility assays on plants pre-treated with the egg extract. (**A**) Feeding assay where leaf damage was automatically quantified by means of machine learning four days after mite infestation. (**B**) Total amount of eggs produced by females on treated or non-treated leaves. (**C**), Number of eggs laid by the mites on the leaf (blue) and outside of the leaf (orange). Significance was determined in (**A**) and (**C**) using a GLM and pair-wise comparison with Bonferroni correction (p < 0.05); and in (**B**) using a One-Way ANOVA followed by a Tukey–Kramer test. The control (non-treated) plants are identified by a ‘C’ and the plants pre-exposed to the egg extract by an ‘E’, followed by the hours the samples were exposed to the egg extract previous to the infestation of adult mites. Boxes represent 50% of the information and whiskers 25%; means are expressed as lines and medians as crosses (n = 12–14 in (**A**) and n = 6–8 in (**B**,**C**)). Data were obtained from *A. thaliana* Col-0 genotype, infested with 20 spider mite adults for 4 days.

To determine if the transcriptomic alterations induced by egg extract treatment had a significant impact on the plant defence response to *T. urticae* infestation, a phenotypic damage assay was performed. To that end, plants were exposed to the mite egg extract 3, 24, and 72 h, and were then subjected to spider mite infestation for four days. Statistically significant variations were found among the pre-treated plants when the damaged area was measured (χ^2^ = 71.7, p < 0.001). Plants previously exposed to the egg extract either for 3 or 24 h did not have any significant effect on plant damage after spider mite infestation. However, a significant increase in plant damage was detected on plants that were previously exposed to the egg extract for 72 h (Fig. 5A).

The potential effect of plants exposed to the egg extract on female fertility was likewise assessed. During this test, plants were also treated with the egg extract for 3, 24, and 72 h. The number of eggs deposited on leaves that had been previously exposed to the egg extract was significantly lower compared to its controls for all time points (F = 22.9, p < 0.001) (Fig. 5B).

A peculiar phenomenon occurred as well in the fertility assay regarding the number of eggs laid on-leaf and out-of-leaf. During the counting procedure, eggs could be found either directly on the leaf or on silk found on the plate out of it. It was noted that, on the controls, females laid more eggs on-leaf than out of it, which did not happen on the treated leaves. Therefore, a comparison was performed to test if the differences were statistically significant (*χ*^2^ = 315.6, *p* < 0.001). The post hoc comparison showed that the pattern of eggs being laid on-leaf was altered on treated leaves (Fig. 5C). In addition, the number of eggs laid on-leaf of pre-exposed leaves equalled its off-leaf amount during the first 24 h, but that changed at 72 hpt, when the egg numbers increased. Noticeably, the oviposition off-leaf did not change with time or if there was treatment or not.

## Discussion

Herbivore oviposition constitutes an important event when plants have the opportunity to detect the threat represented by attacking enemies. The activation of defences to deter or to directly damage deposited eggs provides advantages for the plant to prevent future feeding damage. This activation depends on recognising EAMPs, which might be exploited in plant protection to turn on the defence system. However, information on the nature of egg-associated elicitors is scarce, and using crushed egg extracts represent a good method to maximize plant response. The study of this process using model organisms such as *A. thaliana* and *T. urticae* constitutes a valuable source of information mainly due to the vast resources and research tools currently available to analyse the interaction between both species. Additionally, the possibility of extrapolation of the results from *A. thaliana* to species of agricultural interest enhances the spectrum of applicability.

### *T. urticae* egg extract triggers moderate changes in gene expression

Mild transcriptomic responses were expected for the present study due to the observed oviposition behaviour of spider mite females, and the way the eggs physically interact with the plant. *T. urticae* females lay 1–12 eggs per day on beans in a time frame of approximately 20 days at 25 °C in laboratory conditions^29^. Eggs are oviposited directly on the surface of the plant, or on silk, and the oviposition occurs in a dispersed manner, apparently without any pattern. Moreover, no substance has been described so far that binds or glues the eggs on plants out of silk, which indirectly serves that purpose. This behaviour could represent an evolutionary adaptation, in which the interacting surface between egg and plant is diminished, so the plant has fewer chances of detecting eggs and the new mite generations would have better chances of surviving on a “poorly-informed” host. However, *T. urticae* oviposition differs from other arthropods, such as pierid butterflies that lay their eggs by forming either aggregated or dispersed clusters which produce a necrotic effect on the plant tissue they are laid on^27^. Consequently, their hosts respond by regulating the expression of hundreds of genes at time points as late as 72 h post laying^30,31^. On the other hand, heteropterans display a wide array of oviposition mode variations^32^, which include laying on the surface as in our case, inserting them into the plant tissue, aggregating them or laying them individually^13^. Plant response to aggressive oviposition includes histomorphological alterations and many other strategies involving wide transcriptomic alterations^1,2,5,30,31,33^. Plants affected by spider mite eggs do not present a visible response. A similar result was observed when the butterfly *Aglais io* oviposited on Arabidopsis^34^. *A. io* belongs to the Nymphalidae family, a close relative to the pierids, although unlike them, they do not usually feed on brassicas. Griese et al.^34^, suggested that the plant recognition and response to the oviposition of its frequent pierid foes might be the result of a long evolutionary process. *A. thaliana* plants exposed to spider mite egg extract did not show cell death; however, accumulation of callose and hydrogen peroxide was observed. Opposite to our results with the TB assay, both pierid oviposition and egg extract application produced localised cell death^9,30^. However, in terms of callose deposition and peroxide production, the patterns of the bursts were similar for the latter ^9^ and localised as occurred in the former^30^.

Another interesting point relates to the close relationship existing between oviposition and feeding in our model. Unlike lepidopterans, which oviposit and then larvae feed on the plant, the spider mite feeding stress occurs concurrently with the oviposition process. This means that both stresses are tightly linked in nature, and plants are more likely to accurately respond to the process as a whole than to each of them individually, due to potential synergistic procedures. By isolating oviposition from other processes perceived simultaneously, the plant is expected to react moderately, as if having only a set of pieces of the whole puzzle. This situation has been tested with heteropterans, whose females usually feed while ovipositing. A combination of oviposition and feeding on the aforementioned insects induces in plants the production of volatile blends that differ from the ones produced when both stresses are separated^13^. A study that would further support the hypothesis of the *“*partial stress*”* would be the one performed by^15^. In this study, a transcriptomic profile of the early moments of spider mite infestation on plants was performed. As feeding females also oviposit, defecate, and mechanically stimulate the plant, this constitutes a perfect example of the full biological stress and its transcriptional response of the plant. As expected, the response of the plant involved thousands of genes, whereas in our case, the response only involved hundreds. The log_2_FC of the response in the previously mentioned study was more than two times higher than in ours for the upregulated genes, and near two times higher for the downregulated ones. Similar response mechanisms were found for both sets of genes, where JA cascades, callose deposition, Ca^2+^, ROS, and glucosinolate responses were some of the most relevant. The functional response to the feeding study also was, as expected, richer and more abundant. The similarity and expected differences between the two studies offer insight to the complementarity that exists when the two stresses occur simultaneously, suggesting possible synergistic interactions, as mentioned above.

### Earlier events rely on Ca^2+^ and ROS levels

The highest absolute number of genes was detected as regulated at the earliest time point (3 hpt), which also had the highest levels of up- and down-regulated genes. This information suggests an early transcriptomic “explosion” in the plant, that involved several metabolic cascades, including defence-related pathways. At the first time point evaluated (3 hpt), the earliest response identified was related to Ca^2+^ and ROS processes. Among the induced genes were some that code for NADPH oxidases located at the plasma membrane, such as *RBOHC* and *RBOHD*, which are the main generators of extracellular O^2·−^^5^. These oxidases have been associated with responses to abiotic and pathogen stresses^35^. The extracellular ROS would penetrate the cell by means of aquaporins, but would also activate importing Ca^2+^ channels, further increasing its internal concentration^36,37^. Influencing the intracellular calcium concentrations, the exporting Ca^2+^ channel ACA8 was significantly downregulated. ACA8 is a Ca^2+^ pump located at the plasma membrane whose function is to control intracellular Ca^2+^ levels^38^, associated with defensive activities^39^. The alteration of the aforementioned genes is linked to the increase of internal Ca^2+^ and ROS concentrations, triggering genes activated by those signals. Supporting this statement, a plethora of ROS-associated genes were regulated. Some of them were genes encoding heat shock proteins like HSP17.6B and HSP17.6II, associated with oxidative stress^40–42^ and HSP70, involved in defence responses^43,44^. Also activated were a set of glutathione transferases associated with HAMP responses (GSTF7 and GSTF6)^45,46^, or glucosinolate-dependent activated and associated with responses to H_2_O_2_ (GSTU4)^47^. In addition, the oxidative stress responding gene MSRB7 was altered^48^, which is involved in detoxification^49^.

### JA operates as the main signalling pathway

Most of the genes participating in the α-linolenic acid metabolism were upregulated at the earliest time of sampling (3 hpt). A JA defence response tied to Ca^2+^ level alteration is a common feature of plants to egg elicitors^5,50^. A possible link between the Ca^2+^ and JA cascades in our assays could be the activated calmodulins CML37 and CML50. The upregulated *CML37* is known to respond to stimuli relating to herbivory and has a role as an intermediary between Ca^2+^ and JA cascades^51,52^. Many JA-dependent genes were likewise regulated at 3 hpt. Some of the most relevant include the activation of *MYC2* and its dependent gene *VSP2* that serves as a marker of herbivory^53^. In addition, JA-dependent genes responsible for the production of volatiles were activated, the most relevant being *BSMT1*, *TPS10,* and *CYP82G1*. The two latter genes are responsible for the biosynthesis of monoterpenes and the diterpene TMTT, respectively^54,55^, and *BSMT1* participates in the methylation of SA and benzoic acid^56^. Interestingly, these genes are related to volatile blends produced by lima beans (*Phaseolus lunatus*) when exposed to *T. urticae* and participate in the attraction of the predatory mite *Phytoseiulus persimilis*^57^. Some of the genes activated during this first time point participate in the transformation of JA and JA-Ile to inactive forms (Fig. S8). This means that at this time point the JA pathway had its negative feedback system activated. Among the genes transforming JA-Ile are the cytochrome P450 *CYP94C1*, which produces the derivate 12-COOH-JA-Ile^58^, the genes *JAO2*, *JAO3* and *JAO4* that produce 12-OH-JA^59,60^, and the gene *ST2A* that catalyses the sulfation of 12-OH-JA^61^. Aside from hydrolysing JA-Ile, *IAR3/ILL4,* and *ILL6* are also responsible for transforming 12-OH-JA-Ile into 12-OH-JA^62,63^. Moreover, the activation of the genes *JAZ2*, *JAZ6* and three transcripts of *JAZ9* reinforce this feedback regulation. *JAZ* genes are negative regulators of the JA pathway, being repressed at the start of its cascade, then activated as a negative feedback system when the JA levels are high^64,65^. Linking JA response to final defensive responses, increasing PAD3 expression and camalexin levels have been reported in response to egg exposure in *A. thaliana* plants carrying eggs from the lepidopteran *P. brassicae*^66^. The absence of PAD3 deregulation upon *T. urticae* egg treatment suggests an insignificant contribution of camalexin in the plant response. Finally, glucosinolate production has been broadly associated with Arabidopsis resistance to *T. urticae*^24^. Deregulation of several genes involved in glucosinolate production from methionine and tryptophan, such as *SOT16*, *BCAT4,* and *MAM1* were detected.

### Defence response continues via JA/ET pathway

**Figure 6:**
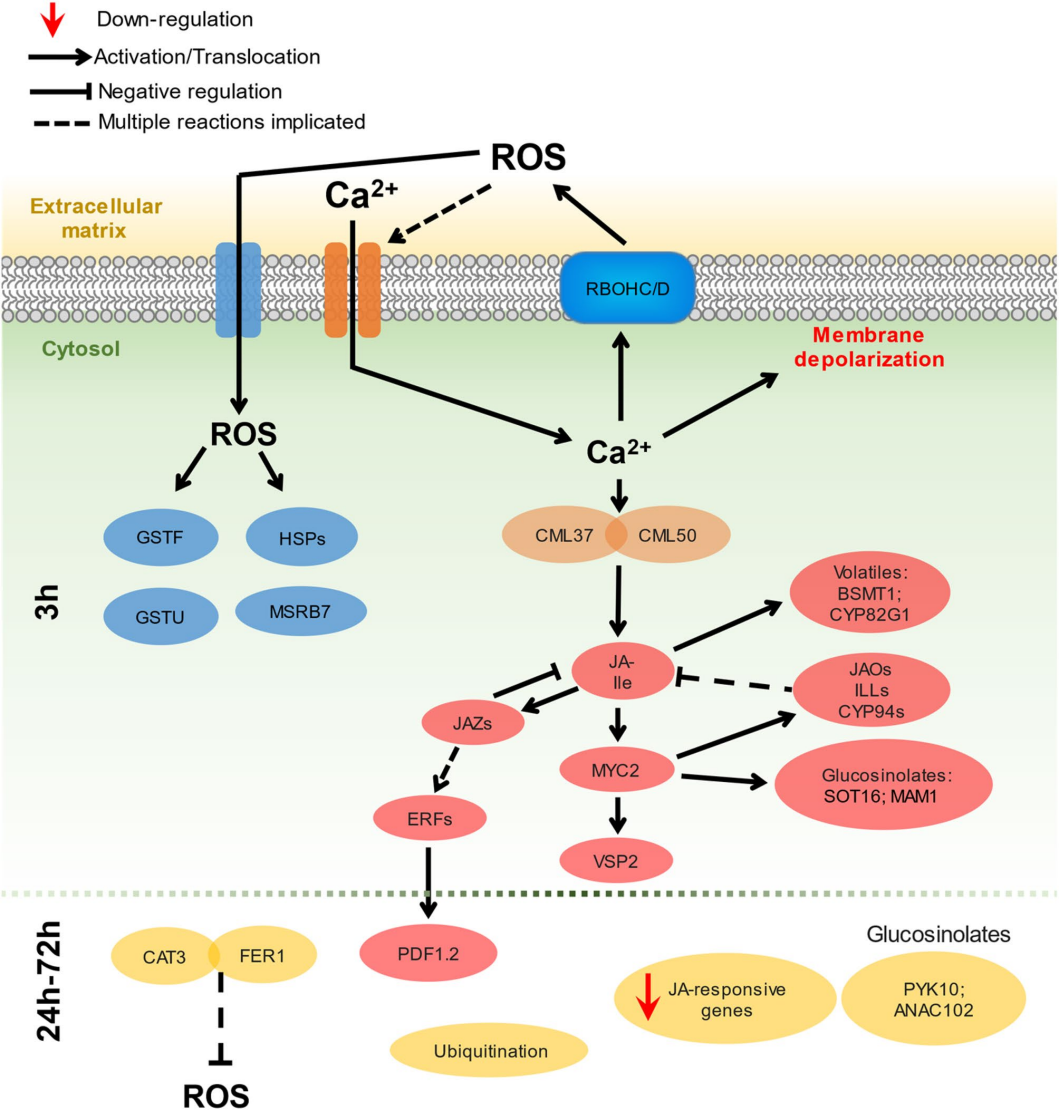
Main pathways during the response of *A. thaliana* to *T. urticae* egg extract. Events were constructed based on transcriptomic evidence. Ca^2+^ imbalances (orange) and ROS production (blue) are known processes preceding JA cascades (red); later events (24–72 hpt) are represented in yellow colour. A plausible model would be as follows: Extracellular alterations would induce the intracellular release of Ca^2+^, which produces membrane potential alterations and the activation of RBOHs. Extracellular ROS produced by the RBOHs, would enter the cell via aquaporins (blue) and open Ca^2+^ channels (yellow), increasing intracellular Ca^2+^ and ROS concentrations. Multiple genes would respond to Ca^2+^ and ROS altered levels (orange and blue respectively). CMLs would activate JA cascades and their associated genes (red), with the activation, first of the MYC2-dependent branch and posteriorly the PDF1.2-dependent branch in the JA-ET crosstalk. Later events include scavenging processes, ubiquitinations and glucosinolate-associated processes. Round boxes indicate Plasma Membrane (PM)-associated proteins, ellipses indicate genes and a red arrow indicates down-regulation.

Now, if JA cascades are coming to an end and its core components indicate a switch to another hormonal pathway, the question of which cascade is replacing JA arises. A schematic representation of the molecular events triggered by the mite egg extract offers cues to temporal transcriptional switches (Fig. 6). The induction at 3 hpt of the ethylene-responsive factors (ERF) *ERF056*, *ERF4,* and *ERF1A* suggests a shift towards the ethylene pathway^67^. The strongest evidence of ET involvement becomes evident when at 24 hpt *PDF1.2* and *PR4* are activated. The induction of *PDF1.2* and *PR4* is controlled by ERFs and is a hallmark of the integration of the JA and ET pathways^68,69^. JA cascades interact with ET cascades by means of two branches^67^. The first branch, that inhibits *JAZ* genes (maintaining the inhibition of the ET pathway), activates plant defence by means of *MYC2* and *VSP2*. The second branch uses JAZ, which would induce the activation of ERFs and consequently *PDF1.2*. Our data suggest that at earlier time points, Arabidopsis responds by means of the *MYC2* and *VSP2* branch, and then switches to the second branch, maintaining *PDF1.2* and *PR4*-based defences at least up to 24 hpt. On the other hand, homeostatic processes are significantly modified at 24 hpt, probably to restore ion and redox imbalances. For example, the expression of the genes encoding the transporter FER1 and the catalase CAT3 was upregulated and these proteins have been associated with ion homeostasis and ROS detoxification^70,71^. Besides, several JA-responsive genes were now downregulated, such as the aminotransferase *TAT3*, the sulfotransferase *SOT17,* and the protein *NSP1*.

Transcriptional reprogramming continued at 72 hpt, represented by a set of factors ERFs related to stress processes. At5g61590/ERF107 and RAP2.4 have been associated with cuticular wax biosynthesis^72,73^, and RAP2.4 and At1g22190/RAP2.13 regulate the synthesis of aquaporin genes^74^. Besides, the genes encoding the enzyme PYK10^75^ and the transcription factor ANAC102^76^, linked to glucosinolate metabolism, were found upregulated, as well as the ubiquitin ligases At1g80440/KMD1 and At3g59940/KMD4 that control phenylpropanoid biosynthesis and cytokinin sensitivity^77^. In addition, the activation of the RNA-dependent RNA polymerase 6 (*RDR6*) at 72 hpt suggests a redirection of stress-induced networks towards their “basal state”. *RDR6* is a regulator of the immune response controlling the mRNA levels of the NLR receptors at post-transcriptional level. When pathogen challenge occurs, *RDR6* levels drop to allow the appropriate response and then increase to produce a “robust silencing” of the defence response^78^.

### Oviposition is diminished in plants pre-exposed to the extract up to 24 h

During the phenotyping assay, the results indicated a peculiar set of events in motion. Due to the presence of classical defence mechanisms such as JA and JA/ET cascades*,* the expectation would be an impact on both feeding and oviposition. However, the latter was affected while the former seemed not to be. Oviposition events tend to be an area of divergent results, reporting detrimental implications for the herbivore feeding stage^2,4,79,80^, and enhanced feeding performance and survival^5,8^. Moreover, the complexity of the interaction of the components in a defence response cascade must be taken into account^81^. Thus, alterations in ROS-related genes can lead to significant plant susceptibility to *T. urticae* feeding and oviposition. The results occurred even when defence hallmarks such as *PDF1.2* and *VSP2* presented expression levels many times higher compared to ours. This would mean that there is no simple relationship such as high levels of a particular gene leading to an absolute protection, but a delicate interaction of multiple processes. On the other hand, pre-exposition of plants to the egg extract having a detrimental effect on female fertility has been reported for other herbivores. Austel et al.^82^ described how prior egg deposition of *Xanthogaleruca luteola* on *Ulmus minor* reduced the reproductive capacity of females and increased larval mortality. Indeed, plants pre-exposed to eggs can likewise affect new generations hatching from eggs^33^, being responsible for pupal and larval weight loss and reducing survival.

### After 72 h of pre-exposition, plants are more susceptible to feeding

Even when the phenotyping events in plants pre-exposed up to 24 h seem to differ, as previously discussed, at 72 h a shift seems to happen. When taking an in-depth look at the oviposition behaviour of the females, similarities to the feeding results become more evident. Oviposition was effectively reduced for all plants pre-treated. However, a closer look reveals that the number of eggs directly laid on the surface of the leaf were the ones that suffered the reduction. Female oviposition on silk out of the leaf was not affected at any time point, not even increased, which would suggest a change in ovipositing site preference. Now, the clear link between the events occurring during the feeding and oviposition assays is revealed on plants exposed to the extract for 72 h. On those plants, the egg number laid on the leaf increased over its own off-leaf amount, which represented a departure from the similarity between on-leaf and off-leaf oviposition that occurred at the first two time points. Likewise, an increase of damage performed on that group of plants (pre-treated for 72 h) was observed. The functional analysis of the transcriptomic data also suggested a shift in plant response at 72 hpt. Moreover, the timeline analysis of the DEGs and DETs indicated catabolic processes mediated by ubiquitination and defence suppressing mechanisms mediated by *RDR6*, not present at earlier time points. Summing up the previous information, the evidence might suggest that up to 24 hpt there are several molecular events that do not affect feeding but do affect oviposition. When the plant has been continuously exposed for 72 h to the egg extract, the molecular events shift. From that point, when challenged by the spider mite, the plants seem to be more susceptible to feeding. Moreover, the oviposition on the plant surface increases, even when the total egg number remains significantly lower compared to its control.

Since the transcriptomic response of the plant to the egg extract affected female oviposition, but not plant damage at the first two time points evaluated, further assays could be performed to understand this result. A deep metabolomic analysis would reveal metabolite alterations, given that a classical defence strategy of plants consists of changing the nutritional quality of their tissues^5^. The possible surge of secondary metabolites, protease inhibitors, or the effect of glucosinolates, spotted among the DEGs, would be interesting information to collect that would further clarify these processes. The effect of possible volatile blend changes on female preference or the attraction of predatory mites could be also taken into account. Additional analysis could also be pursued to answer questions such as the potential impact on egg hatching, adult fitness, and survivability of mites grown on plants previously exposed to the egg extract.

## Materials and methods

### Plant material and growth conditions

The ecotype Columbia (Col-0) from *A. thaliana* was used for all experiments. Sterilized seeds were planted in a mixture of peat moss and vermiculite (2:1 v/v) and incubated in the dark for 5 days at 4 °C. Plants were then transferred to growth chambers (Sanyo MLR-350-H) under controlled conditions (23 °C ± 1 °C, > 70% RH and 16 h:8 h day:night photoperiod).

### *Tetranychus urticae* rearing

A colony of *T. urticae*, London strain (Acari: Tetranychidae), kindly provided by Dr. Miodrag Grbic (UWO, Canada), was reared on beans (*Phaseolus vulgaris*) and maintained in growth chambers (Sanyo MLR-350-H, Sanyo, Japan) at 25 °C ± 1 °C, > 70% relative humidity (RH) and a 16 h:8 h day:night photoperiod.

### SEM analysis

A piece of bean leaf containing spider mite eggs and silk was observed by scanning electron microscopy (SEM). Egg images were obtained using Hitachi TM3030Plus (Hitachi High-Technologies Co. Ltd., Tokyo, Japan) operated at a 5 kV accelerating voltage under low vacuum.

### Egg production, collection, and extract preparation

To obtain *T. urticae* eggs, adult females were reared on fresh bean leaves kept inside of ventilated boxes, previously prepared with wet cotton to avoid mites escaping (Fig. S9A). After three days of feeding, females were removed from the boxes and the eggs were isolated by using a vacuum pump connected to an Eppendorf tube, and counted during the process. Any possible contamination was cleaned by using a thin paintbrush, avoiding egg disturbance. Eggs were immediately stored at − 20 °C.

Collected eggs were immersed in MilliQ autoclaved water (about 100 eggs per microliter of water). Eggs were then mechanically ground, centrifuged (1 s, 9000 g), and sonicated (30 s) to obtain the final homogenate (Fig. S9B).

### Histochemical staining

Plant damage was assessed by analysing the accumulation of hydrogen peroxide (H_2_O_2_) and callose and measuring cell death in response to the spider mite egg extract. Three-week-old plants were treated by applying 2 μL of the egg extract on two opposite leaves. Control plants received two microliters of water. After 3, 24, and 72 h of treatment (hpt), the leaves were excised and subjected to DAB to determine H_2_O_2_ accumulation according to^83^. To evaluate callose deposition, leaves were incubated in 95% ethanol and stained with aniline blue according to^84^. Cell viability was analysed by trypan blue staining as described by^85^. All images were visualized using Leica Fluorescence Stereoscope MZ10F and Zeiss Axiophot microscope.

### Plant treatment and RNA extraction

To analyse the transcriptional responses of *A. thaliana* to the spider mite egg extract, the experimental design depicted in (Fig. S9A) was used. Three-week-old plants were treated by applying 2 μL of egg extract as described above. After 3, 24, and 72 hpt, whole rosettes were sampled, immediately frozen in liquid nitrogen, and stored at –80 °C until used for RNA extraction. Three biological replicates of treated and control tissues were collected for each time point. Each biological replicate consisted of a pool of five whole independent rosettes. Frozen material was thoroughly grinded in liquid nitrogen and total RNA was extracted by means of the RNeasy plant mini kit (Qiagen), and a DNase treatment (Qiagen) following manufacturer´s instructions.

### cDNA synthesis and RT-qPCR

First‐strand cDNA was obtained from 2 μg of total RNA in a volume of 10 μL using the Revert Aid H Minus First Strand cDNA synthesis kit for RT-qPCR (Thermo Fisher Scientific). Three technical replicates were performed for each biological replicate for RT-qPCR assays. The aforementioned assays were performed using the LightCycler 480 SYBR Green I Master Mix (Roche), on a LightCycler 480. qRT-PCR conditions used were: 40 cycles of 15 s at 95 °C, 1 min at 55 °C, and 5 s at 65 °C. Each gene´s primer efficiency was tested using a standard curve and target gene specificity was checked using a melting curve. Relative expression was calculated according to^86^ having Ubiquitin as the reference gene and was represented as relative expression level (2^−dCt^) or fold change (2^−ddCt^). Specific primer sequences were taken from previous publications or de novo designed through Primer3Plus^87^. Primer sequences and sources are listed in Table S3.

### Transcriptomics

The quality and integrity of RNA samples were assessed by means of electrophoresis on 1% agarose gels, NanoDrop ND-1000 spectrophotometer (NanoDrop Technologies) and using the Nano 6000 Assay Kit of the Bioanalyzer 2100 system (Agilent Technologies). RNA libraries were generated using NEBNext Ultra RNA Library Prep Kit for Illumina according to manufacturer’s instructions. Libraries were sequenced on an Illumina platform generating paired-end fragments of 150 ~ 200 bp. Raw data obtained in FASTQ format was processed to remove adapters and poly-N sequences as well as low quality reads. DNA sequences were analysed and processed using Trimmomatic (Galaxy Version 0.38.0)^88^, dropping reads below Q15 with a sliding window of 4 bp. Reads whose length was below 108 bp were removed; clean reads were in average 27 million per sample. Quality of the resulting data was assessed by evaluating Q20, Q30, and GC content. Quality reports were produced before and after the Trimmomatic procedure, using the FastQC toolkit^89^. Trimmed reads were aligned to the *A. thaliana* (TAIR10) genome (https://www.arabidopsis.org) using Kallisto^90^. Kallisto was used to calculate the abundance of the transcripts as transcripts per million (TPM) by pseudo-aligning the sample reads to the reference using the default k-mer length of 31 and 100 bootstrap repetitions. Fold changes (FC) and adjusted *p*-values (*q*-value) for each gene were calculated using the Sleuth software. Each time point was compared to its corresponding control by means of a Wald test and the Lancaster aggregation method^91^. The Lancaster process was selected based on its capacity to outperform classical gene-level analysis, such as Šidák, and because it has greater power at lower FDR^92^. The aforementioned procedures were based on the normalised measure of gene abundance: Transcripts Per Million (TPM) were calculated by Kallisto. TPMs are reported as superior to previous measures such as RPKM, due to its capacity to avoid inflated statistical significance, improve accuracy, and avoid inter-sample artificial differences in RNA abundance^92,93^. Differentially expressed transcripts (DETs) were considered when the false discovery rate (FDR) was < 0.05 and the |log_2_(Fold Change)| was ≥ 0.5; genes were considered as differentially expressed (DEGs) when its Lancaster aggregated *p*-value was p < 0.05. Venn diagrams to depict the DEG lists obtained for each time point were constructed with the InteractiVenn online resource^94^.

### Functional analysis

To analyse the functions and processes activated in response to the egg extract treatment, gene ontology (GO) enrichment analysis was performed based on the DEGs identified. GO analysis was done using the g:Profiler web server^95^, excluding the electronic annotations. Three GO categories were analysed: molecular function (MF), cellular component (CC), and biological process (BP). The aforementioned information produced in g:Profiler was afterwards introduced in Cytoscape for further analysis according to^96^. The EnrichmentMap app^97^ from Cytoscape was used to visualise as a network/map the GO results obtained for each timepoint. To that end, GO terms, DEG and DET lists, expression information, and rankings of the genes, based on *q*-values performed in R^98^, were introduced into the software. FDR *q*-value cut-off parameter was set to < 0.05; the lists of DEGs and DETs were processed independently. To identify clusters of similar terms representing major biological processes, the AutoAnnotate app from Cytoscape used the networks formed by the EnrichmentMap app. For a more detailed analysis, gene enrichment was performed using the ClueGO package v2.5.7^99^ in Cytoscape. ClueGO identified the significantly enriched GO terms and placed them into a functionally organized non-redundant gene ontology network based on the following parameters: min. GO level = 3; max. GO level = 8; min. number of genes = 3–5; min. percentage = 2.0–6.0; GO fusion = true; sharing group percentage = 40.0; merge redundant groups with > 40.0% overlap; kappa score threshold = 0.4; and evidence codes used [All]. To further analyse the specific enzymes and pathways involved in the response of the plant to the spider mite egg extract, information was obtained using the KEGG and PlantCyc databases^100,101^.

### Plant damage estimation

Three-week-old Arabidopis plants were subjected to two consecutive biotic stresses. First, the egg extract was applied as described above, and after 3, 24, and 72 h of treatment infestation with adult *T. urticae* females was carried out. Infestation took place by applying 20 adult females of *T. urticae* per plant. After 4 days of infestation, spider mites were removed from the plants and the entire rosettes were excised and scanned using a size reference on a HP Scanjet (HP Scanjet 5590 Digital Flatbed Scanner series). The scanning conditions and damaged area estimation procedure using Ilastik^102^, occurred according to^103^. Replicates to estimate damage ranged from 11 to 14 plants.

### Female fecundity

Fecundity bioassays were performed on excised whole leaves of *A. thaliana* Col-0. Three-week-old plants were exposed to the spider mite egg extract as described above for 3, 24, and 72 h. Then, a single treated leaf from each of the rosettes was excised and put in the mentioned closed system. Isolated leaves were then infested using 12 synchronised adult spider mite females. After 36 h of infestation, females were gently removed from the leaves and the eggs laid were counted. Experiment conditions were maintained at 25 ± 1 °C, > 70% RH, and 16 h:8 h day:night photoperiod. Six to eight replicates were used for each time point.

### Statistical analyses

GO analyses performed with the g:Profiler web server and with the ClueGO tool used the lists of DEGs and DETs ranked by log_10_(p_adj._). The analysis used the Benjamini–Hochberg value of FDR < 0.05 or the Bonferroni step-down test to determine ontology significance. Egg counting data from the fertility assay were compared using a One-Way ANOVA, differences were then located using a Tukey–Kramer test. Damaged areas estimated on whole rosettes and egg counting data related to the laying location were compared by means of a Generalized Linear Model (GLM), using normal distribution and an identity link function. Post hoc analysis was performed by applying a pairwise comparison analysis with Bonferroni correction. All GLM tests used a Chi-Square distribution to determine differences. The signification was established at p < 0.05 for all tests. All data analysis was performed using the R software version 3.5.3^98^.

### Plant material declaration

Experimental research involving plant material complied with relevant institutional, national and international guidelines and legislations. Seed collection occurred according to relevant permissions.

## Supplementary Information

Supplementary Information 1.

Supplementary Information 2.

Supplementary Information 3.

Supplementary Information 4.

Supplementary Information 5.

## Data Availability

All relevant supporting data sets are included in the article and its supplemental files. RNAseq data have been deposited in GEO under accession code GSE168993.
