# Supplementary material for: Spider mite egg extract modifies Arabidopsis response to future infestations
Source: Sci Rep. 2021 Sep 6;11:17692. doi: 10.1038/s41598-021-97245-z (PMC8421376; doi:10.1038/s41598-021-97245-z)
Supplement: Supplementary file 1 — Supplementary Information 1. [file 41598_2021_97245_MOESM1_ESM.pptx]

## Slide 1
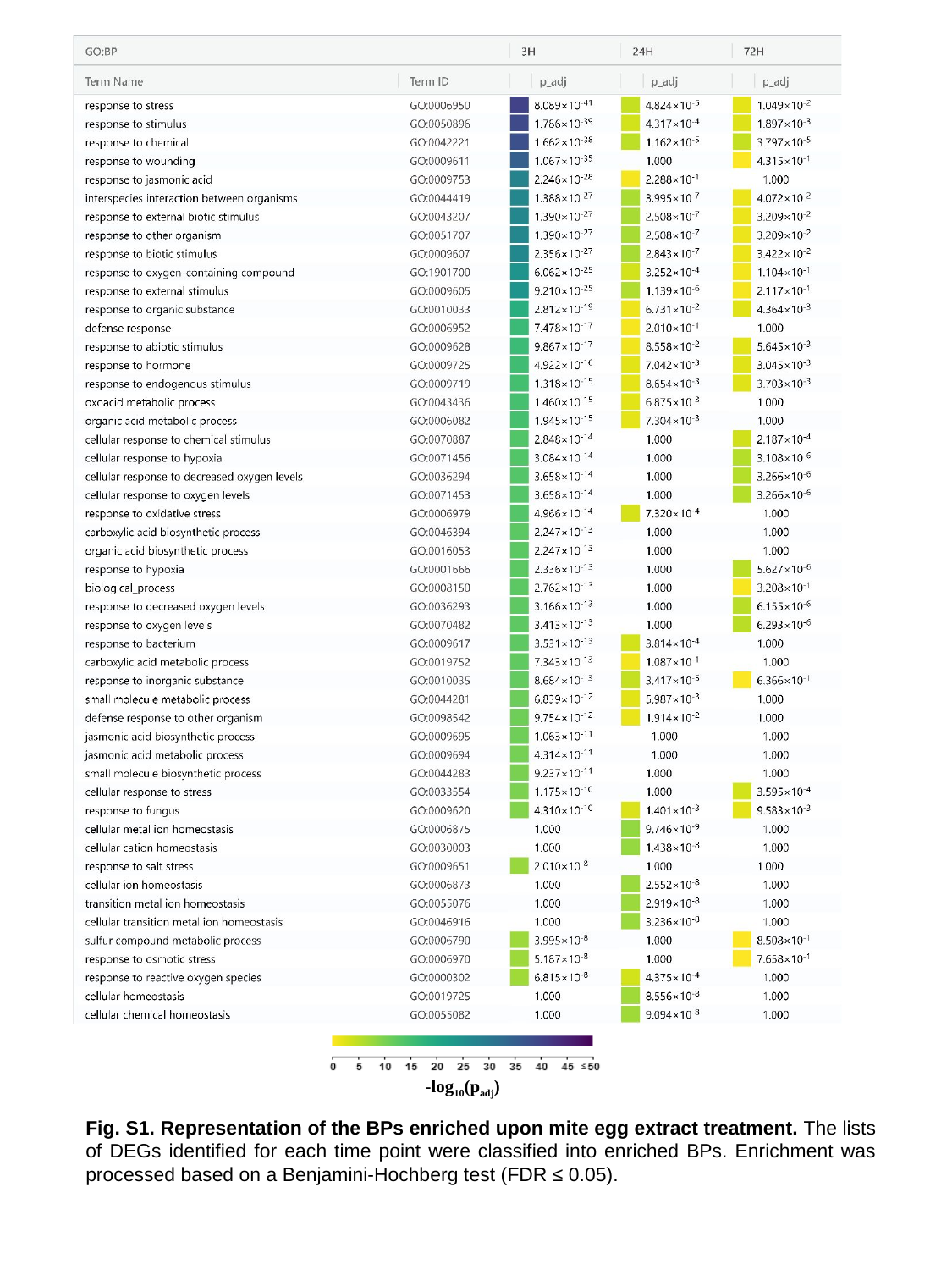

-log10(padj)
Fig. S1. Representation of the BPs enriched upon mite egg extract treatment. The lists of DEGs identified for each time point were classified into enriched BPs. Enrichment was processed based on a Benjamini-Hochberg test (FDR ≤ 0.05).

## Slide 2
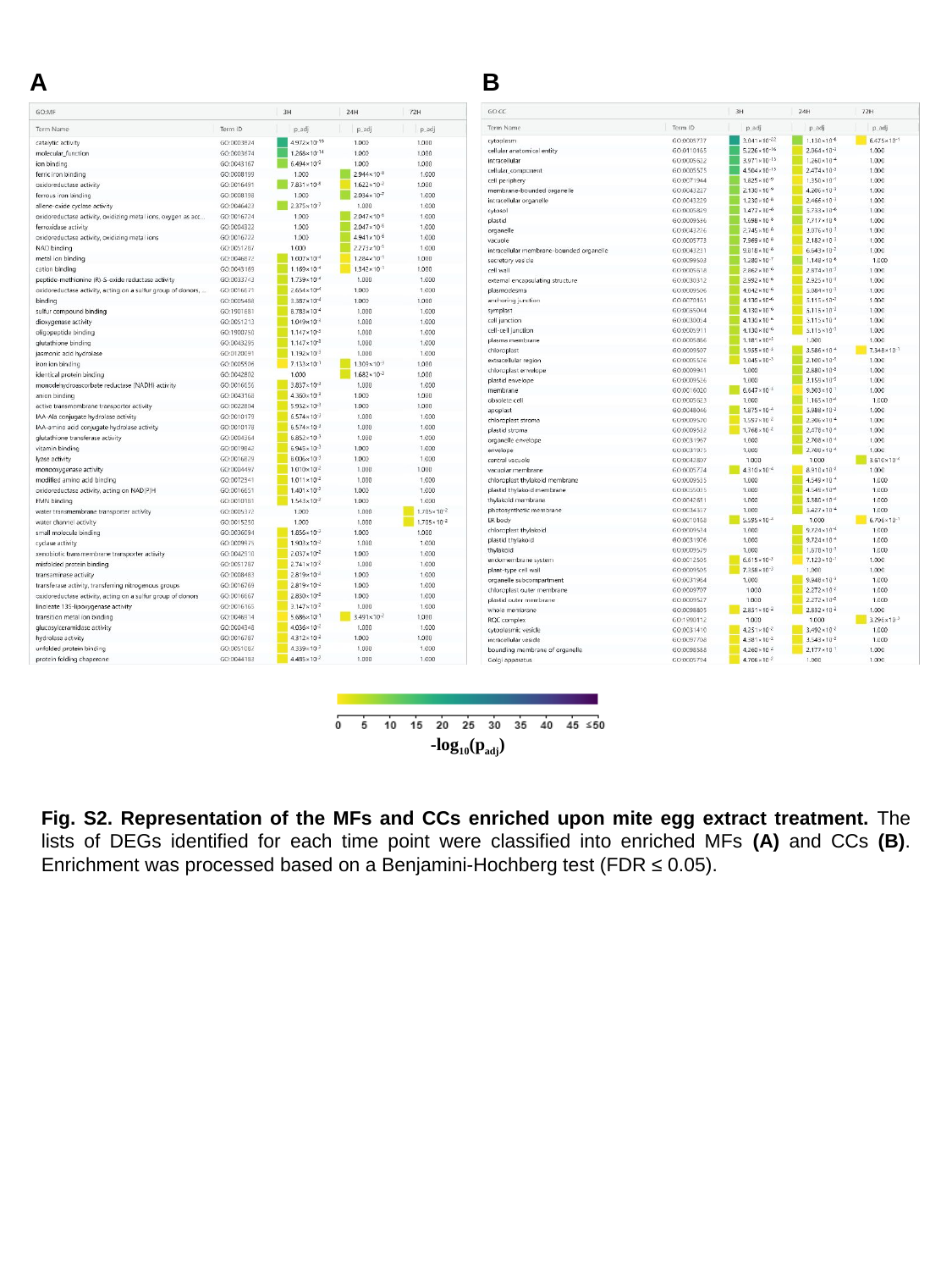

A
B
-log10(padj)
Fig. S2. Representation of the MFs and CCs enriched upon mite egg extract treatment. The lists of DEGs identified for each time point were classified into enriched MFs (A) and CCs (B). Enrichment was processed based on a Benjamini-Hochberg test (FDR ≤ 0.05).

## Slide 3
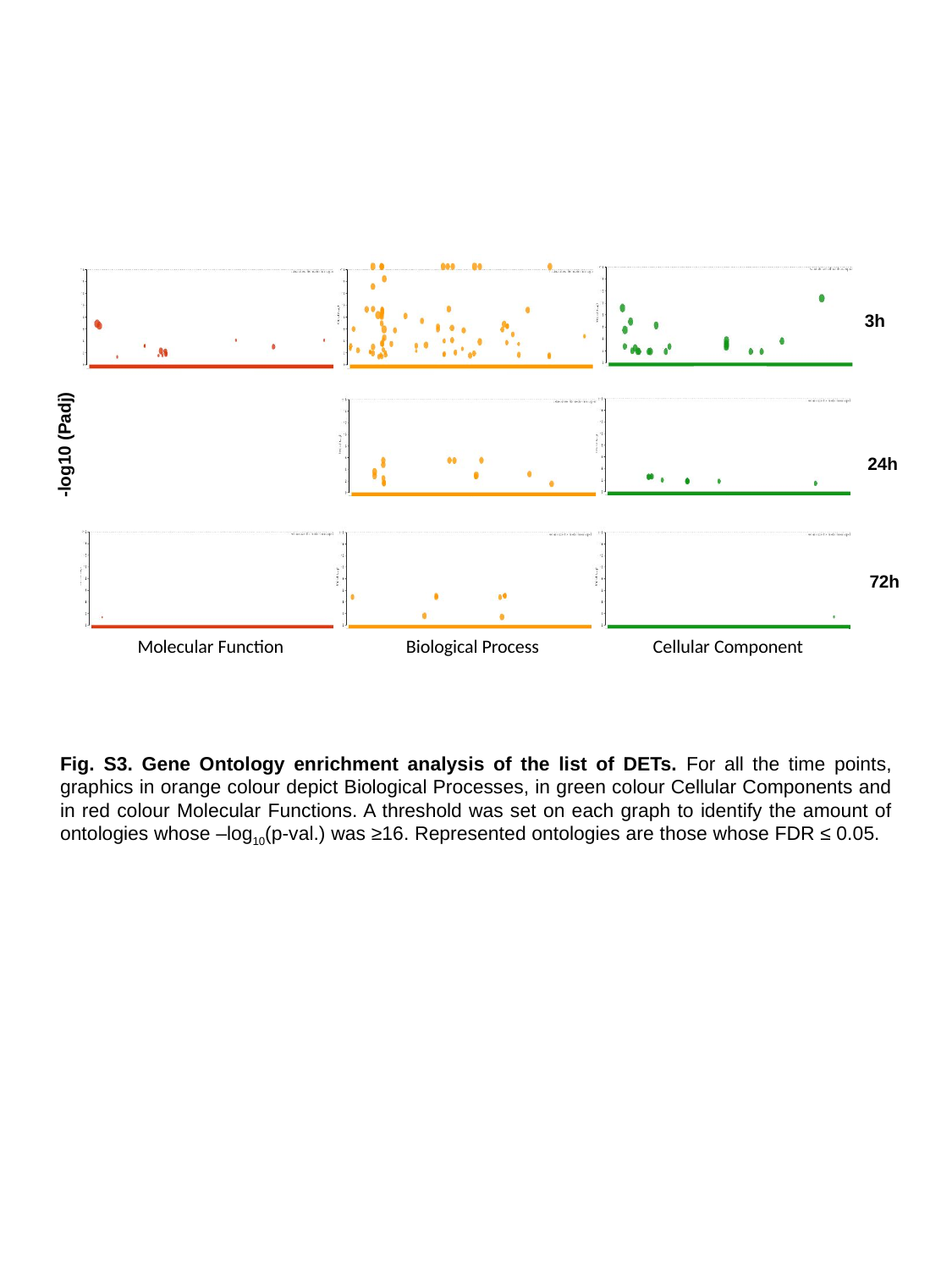

3h
-log10 (Padj)
24h
72h
 Molecular Function Biological Process Cellular Component
Fig. S3. Gene Ontology enrichment analysis of the list of DETs. For all the time points, graphics in orange colour depict Biological Processes, in green colour Cellular Components and in red colour Molecular Functions. A threshold was set on each graph to identify the amount of ontologies whose –log10(p-val.) was ≥16. Represented ontologies are those whose FDR ≤ 0.05.

## Slide 4
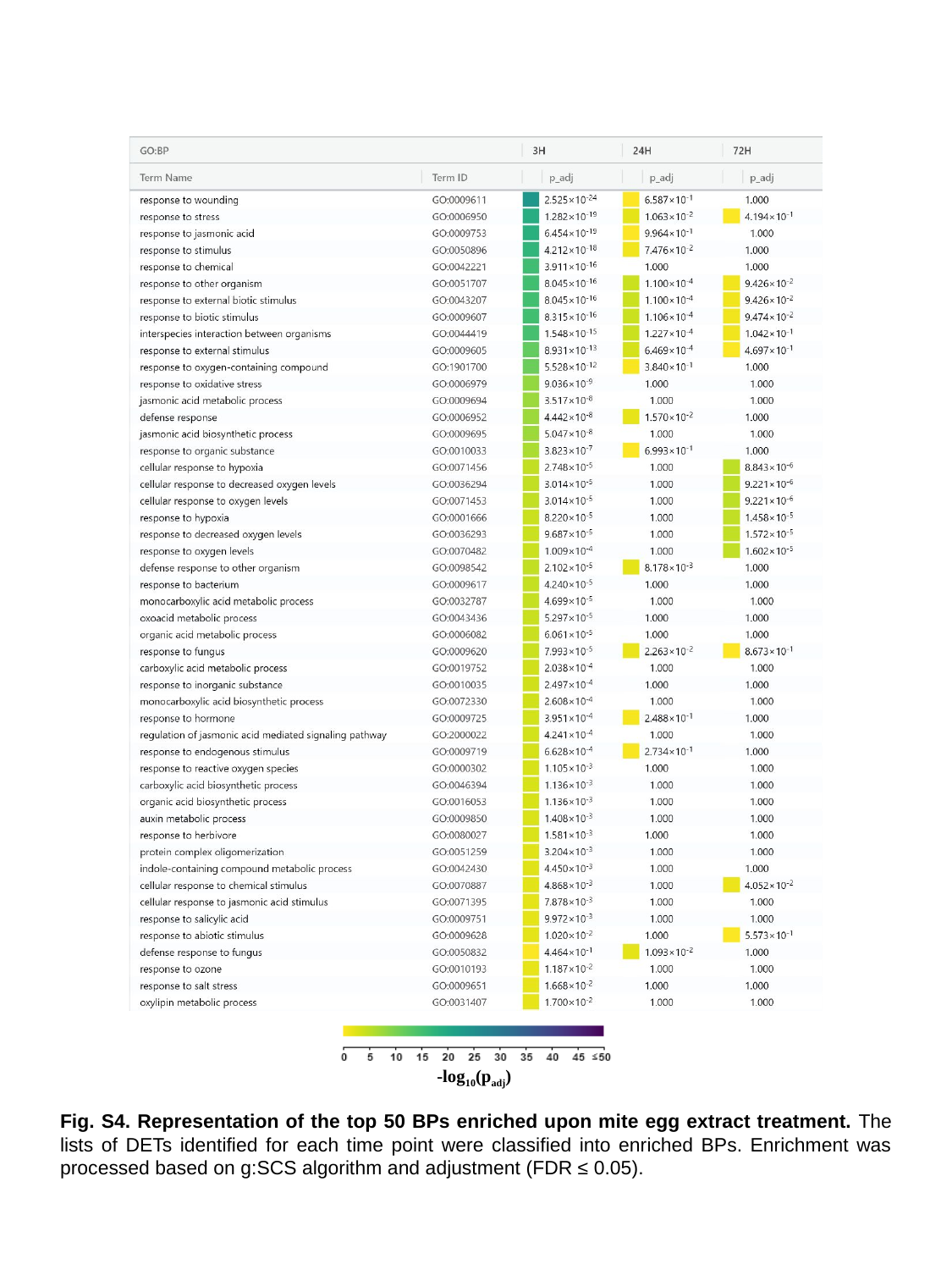

-log10(padj)
Fig. S4. Representation of the top 50 BPs enriched upon mite egg extract treatment. The lists of DETs identified for each time point were classified into enriched BPs. Enrichment was processed based on g:SCS algorithm and adjustment (FDR ≤ 0.05).

## Slide 5
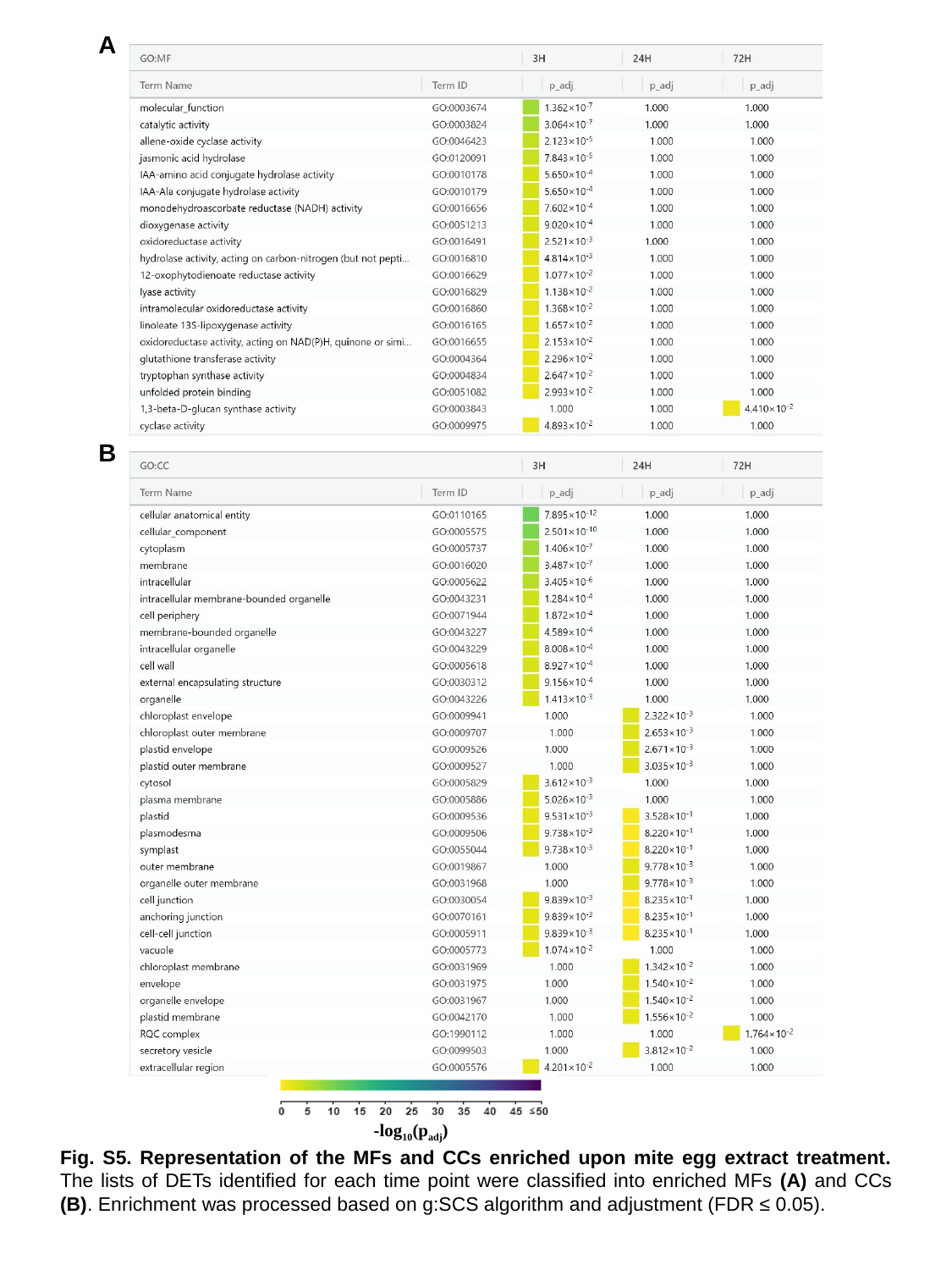

A
B
-log10(padj)
Fig. S5. Representation of the MFs and CCs enriched upon mite egg extract treatment. The lists of DETs identified for each time point were classified into enriched MFs (A) and CCs (B). Enrichment was processed based on g:SCS algorithm and adjustment (FDR ≤ 0.05).

## Slide 6
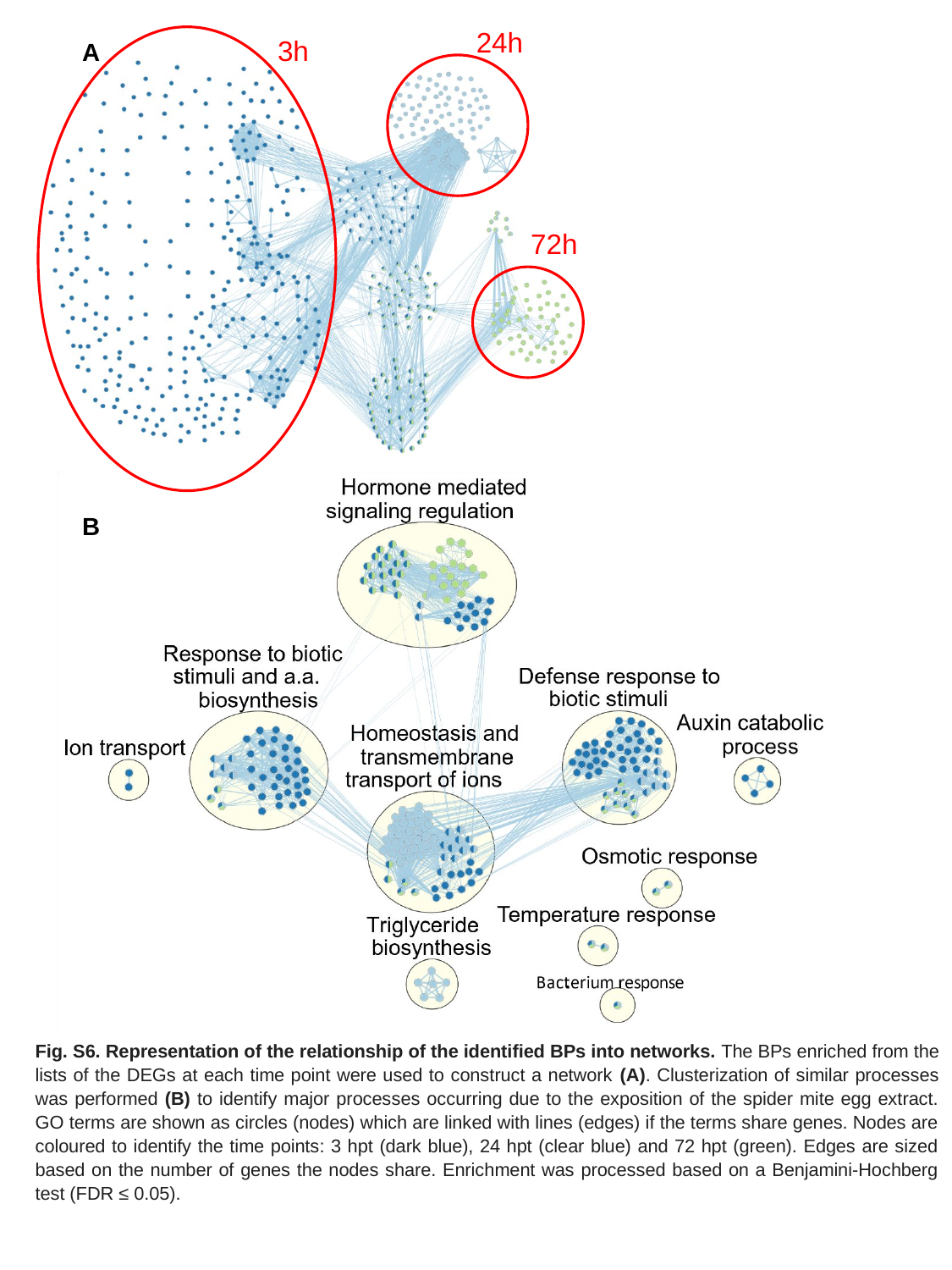

24h
3h
72h
A
B
Fig. S6. Representation of the relationship of the identified BPs into networks. The BPs enriched from the lists of the DEGs at each time point were used to construct a network (A). Clusterization of similar processes was performed (B) to identify major processes occurring due to the exposition of the spider mite egg extract. GO terms are shown as circles (nodes) which are linked with lines (edges) if the terms share genes. Nodes are coloured to identify the time points: 3 hpt (dark blue), 24 hpt (clear blue) and 72 hpt (green). Edges are sized based on the number of genes the nodes share. Enrichment was processed based on a Benjamini-Hochberg test (FDR ≤ 0.05).

## Slide 7
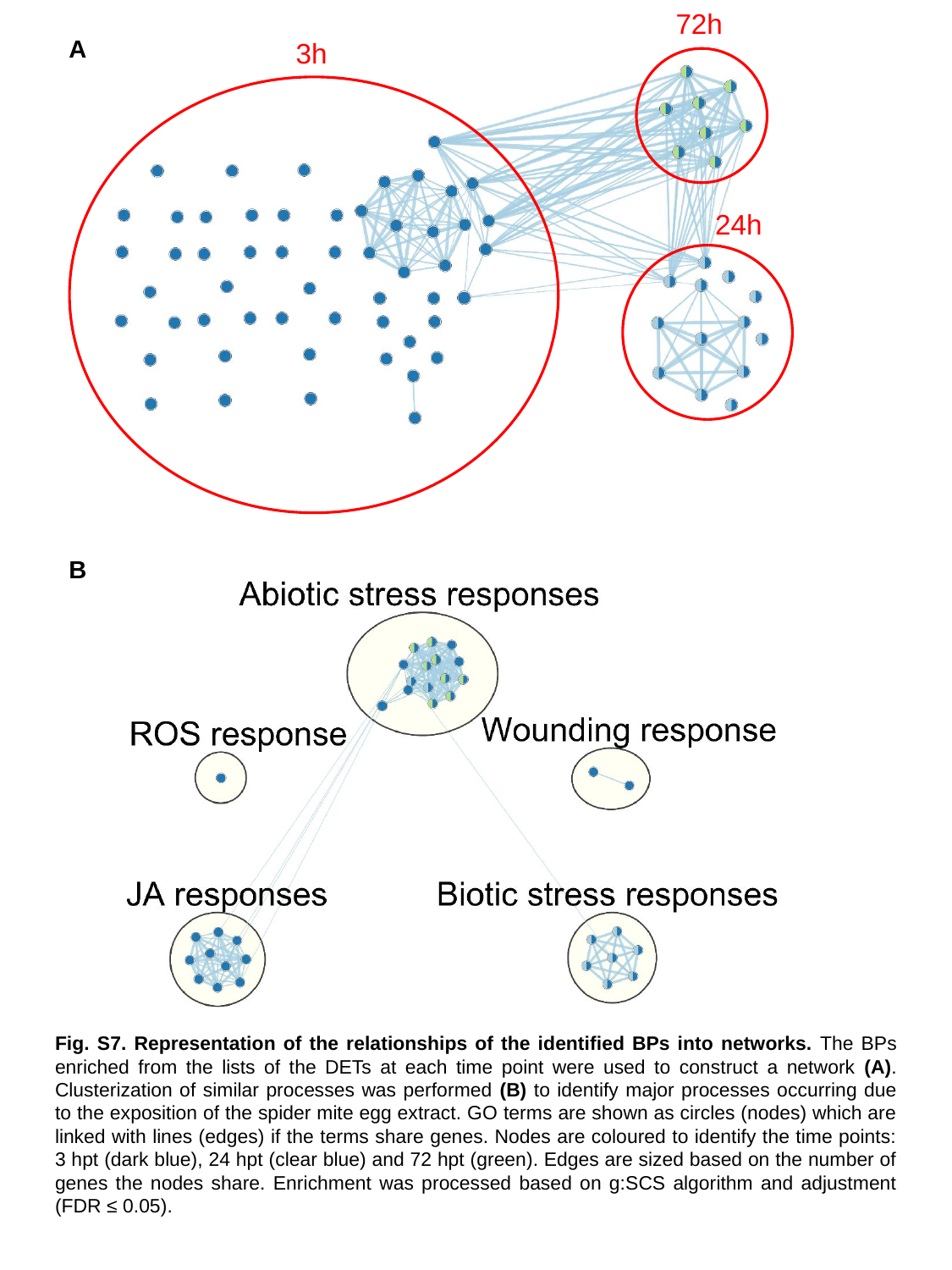

72h
A
3h
24h
B
Fig. S7. Representation of the relationships of the identified BPs into networks. The BPs enriched from the lists of the DETs at each time point were used to construct a network (A). Clusterization of similar processes was performed (B) to identify major processes occurring due to the exposition of the spider mite egg extract. GO terms are shown as circles (nodes) which are linked with lines (edges) if the terms share genes. Nodes are coloured to identify the time points: 3 hpt (dark blue), 24 hpt (clear blue) and 72 hpt (green). Edges are sized based on the number of genes the nodes share. Enrichment was processed based on g:SCS algorithm and adjustment (FDR ≤ 0.05).

## Slide 8
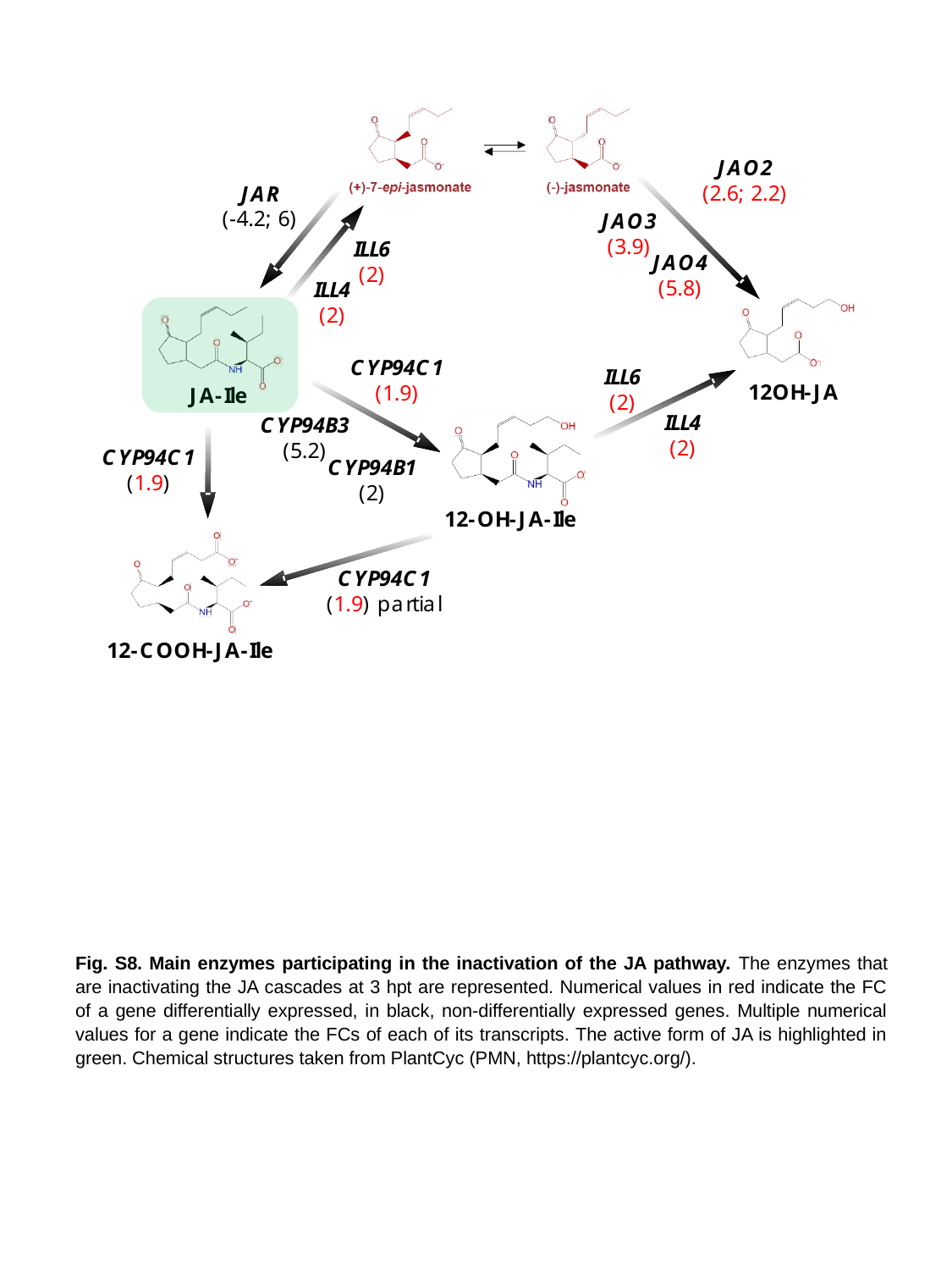

Fig. S8. Main enzymes participating in the inactivation of the JA pathway. The enzymes that are inactivating the JA cascades at 3 hpt are represented. Numerical values in red indicate the FC of a gene differentially expressed, in black, non-differentially expressed genes. Multiple numerical values for a gene indicate the FCs of each of its transcripts. The active form of JA is highlighted in green. Chemical structures taken from PlantCyc (PMN, https://plantcyc.org/).

## Slide 9
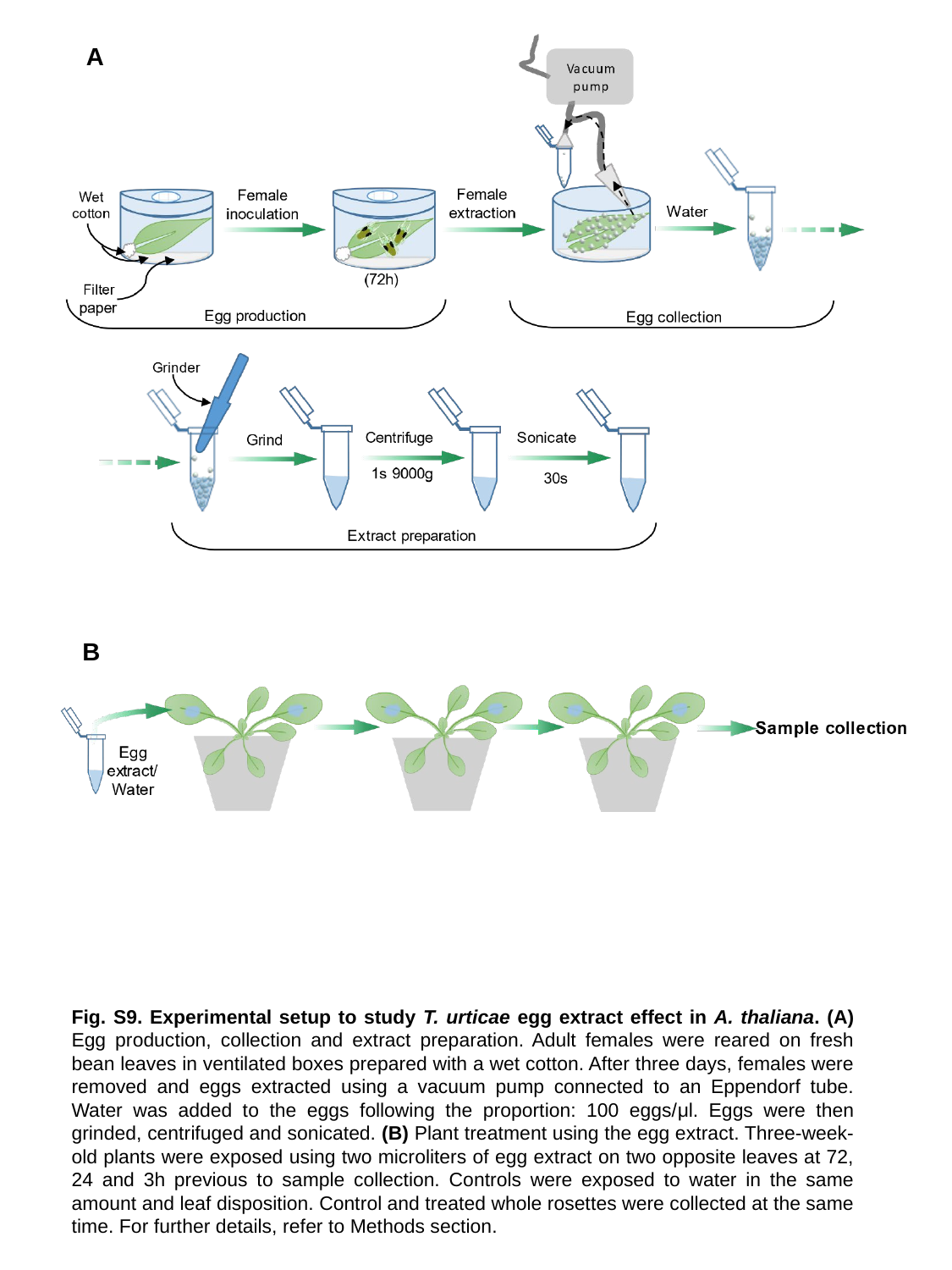

A
B
Fig. S9. Experimental setup to study T. urticae egg extract effect in A. thaliana. (A) Egg production, collection and extract preparation. Adult females were reared on fresh bean leaves in ventilated boxes prepared with a wet cotton. After three days, females were removed and eggs extracted using a vacuum pump connected to an Eppendorf tube. Water was added to the eggs following the proportion: 100 eggs/μl. Eggs were then grinded, centrifuged and sonicated. (B) Plant treatment using the egg extract. Three-week-old plants were exposed using two microliters of egg extract on two opposite leaves at 72, 24 and 3h previous to sample collection. Controls were exposed to water in the same amount and leaf disposition. Control and treated whole rosettes were collected at the same time. For further details, refer to Methods section.
