# Supplementary material for: Spider mite egg extract modifies Arabidopsis response to future infestations
Source: Sci Rep. 2021 Sep 6;11:17692. doi: 10.1038/s41598-021-97245-z (PMC8421376; doi:10.1038/s41598-021-97245-z)
Supplement: Supplementary file 2 — Supplementary Information 2. [file 41598_2021_97245_MOESM2_ESM.pdf]

Table S1 Overview of data production quality

Sample	Raw Read Count	Filtered Read Count	Clean Data(G)	Error Rate(%)	Q20(%)	Q30(%)	GC Content (%)
E_3h_R1	26770534	26361892	7.9	0.02	98.29	94.72	46.25
E_72h_R3	36406131	35873369	10.8	0.02	98.20	94.58	46.27
E_3h_R3	22926911	22630395	6.8	0.02	98.11	94.34	46.09
E_3h_R2	68738552	67857006	20.4	0.02	98.45	95.12	46.27
E_72h_R1	23012045	22724480	6.8	0.02	98.30	94.82	46.28
E_24h_R3	21270464	21048122	6.3	0.02	98.64	95.75	46.40
E_24h_R2	26620946	26513859	8.0	0.02	98.52	95.42	46.35
E_24h_R1	26623469	26516002	8.0	0.02	98.47	95.34	46.32
C_72h_R1	22203351	22033208	6.6	0.02	98.57	95.61	46.37
C_72h_R2	22749188	22633490	6.8	0.02	98.63	95.72	46.32
C_72h_R3	23720614	23423909	7.0	0.02	98.57	95.54	46.29
C_24h_R1	25452898	25269077	7.6	0.02	98.64	95.74	46.38
E_72h_R2	22212891	21872675	6.6	0.03	98.00	94.01	46.31
C_24h_R3	28707444	28458053	8.5	0.02	98.37	95.11	46.37
C_24h_R2	24027530	23916385	7.2	0.02	98.55	95.51	46.45
C_3h_R3	25561886	25204819	7.6	0.03	97.92	93.81	46.14
C_3h_R2	31955022	31631552	9.5	0.02	98.13	94.28	46.25
C_3h_R1	21726222	21379182	6.4	0.02	98.21	94.54	46.26

In the column corresponding to the sample name, names are coded as follows: “C” stands for Control and “E” for Treatment (egg extract), subsequently, the time at which the sample was taken is stated (3h, 24h or 72h); finally, each of the three replicates taken at each time point is identified. The original sequencing read count and filtered read count are represented in columns two and three. The average coverage of the whole genome by the filtered reads is expressed in the fourth column as: Clean read number multiplied by Read length and expressed in genome length (G) unit. The fifth column expresses the average sequencing error as percentage, calculated by: $Q_{phred}=-10\log_{10}(e)$. Sixth and seventh columns represent the Quality Scores, expressing the percentage of correct base recognition over 99% and 99.9% respectively. The eight column expresses the content of Guanine (G) and Cytosine (C) in percentage.

Table S2 Overview of mapping status

Sample name	Total reads	Total mapped	Multiple mapped	Uniquely mapped	Non-splice reads	Splice reads
C_24h_R1	5.1E+07	49899659 (98.74%)	1209607 (2.39%)	48690052 (96.34%)	28445748 (56.29%)	20244304 (40.06%)
C_24h_R2	4.8E+07	47231655 (98.74%)	1484526 (3.10%)	45747129 (95.64%)	26582092 (55.57%)	19165037 (40.07%)
C_24h_R3	5.7E+07	56140803 (98.64%)	1380819 (2.43%)	54759984 (96.21%)	31860039 (55.98%)	22899945 (40.23%)
C_3h_R1	4.3E+07	42182142 (98.65%)	1045755 (2.45%)	41136387 (96.21%)	23943742 (56.00%)	17192645 (40.21%)
C_3h_R2	6.3E+07	62387932 (98.62%)	1563905 (2.47%)	60824027 (96.14%)	35212022 (55.66%)	25612005 (40.48%)
C_3h_R3	5E+07	49643069 (98.48%)	1230389 (2.44%)	48412680 (96.04%)	28117471 (55.78%)	20295209 (40.26%)
C_72h_R1	4.4E+07	43517219 (98.75%)	1090300 (2.47%)	42426919 (96.28%)	24680719 (56.01%)	17746200 (40.27%)
C_72h_R2	4.5E+07	44730647 (98.82%)	1094062 (2.42%)	43636585 (96.40%)	25096458 (55.44%)	18540127 (40.96%)
C_72h_R3	4.7E+07	46323328 (98.88%)	1162274 (2.48%)	45161054 (96.40%)	26249639 (56.03%)	18911415 (40.37%)
E_24h_R1	5.3E+07	52350331 (98.71%)	1272674 (2.40%)	51077657 (96.31%)	29845554 (56.28%)	21232103 (40.04%)
E_24h_R2	5.3E+07	52270010 (98.57%)	1347612 (2.54%)	50922398 (96.03%)	29645162 (55.91%)	21277236 (40.12%)
E_24h_R3	4.2E+07	41560845 (98.73%)	1012008 (2.40%)	40548837 (96.32%)	23575320 (56.00%)	16973517 (40.32%)
E_3h_R1	5.3E+07	52072667 (98.77%)	1237993 (2.35%)	50834674 (96.42%)	29593999 (56.13%)	21240675 (40.29%)
E_3h_R2	1.4E+08	134160199 (98.86%)	3444899 (2.54%)	130715300 (96.32%)	75822525 (55.87%)	54892775 (40.45%)
E_3h_R3	4.5E+07	44600472 (98.54%)	1037652 (2.29%)	43562820 (96.25%)	25149501 (55.57%)	18413319 (40.68%)
E_72h_R1	4.5E+07	44859601 (98.70%)	1047251 (2.30%)	43812350 (96.40%)	25632073 (56.40%)	18180277 (40.00%)
E_72h_R2	4.4E+07	43113345 (98.56%)	1020182 (2.33%)	42093163 (96.22%)	24623865 (56.29%)	17469298 (39.93%)
E_72h_R3	7.2E+07	70821746 (98.71%)	1785912 (2.49%)	69035834 (96.22%)	40077925 (55.86%)	28957909 (40.36%)

In the column corresponding to the sample name, names are coded as follows: “C” stands for Control and “E” for Treatment (egg extract), subsequently, the time at which the sample was taken is stated (3h, 24h or 72h); finally, each of the three replicates taken at each time point is identified. Columns two and three depict the total amount of read obtained and which of them were successfully mapped into the reference genome (TAIR10), respectively. The fourth and fifth columns enumerate the reads that were mapped into multiple sites in the reference genome and uniquely mapped, respectively. The las two columns give the amount of reads that can be mapped entirely to a single exon or mapped to two exons (also named junction reads).

Table S3 Primers used for RT-qPCR validation

Gene abbreviation	ID	Sequence (5' --> 3')	Source
FOX2	AT1G26390.1-F	ATGCCTCAGGTAGCAAATGG	Primer3Plus
	AT1G26390.1-R	CAGGCCAGTTTTGGTTCATT	
ANS	AT2G38240.1-F	GATTGTTTCCGTGCAATCCT	
	AT2G38240.1-R	CAGGGATTTCTATCCCAGCA	
EARLI1	AT4G12480.1-F	CGGTCCCAAGTCCTTCAGTA	
	AT4G12480.1-R	CTGCTTAAAACGTTCGCACA	
MDAR3	AT3G09940.1-F	ACCGCTGAGATAGCTTCATTCT	(Abu-Romman *et al*, 2016)*
	AT3G09940.1-R	CTTCTAGGGTTCTTCCATCCTCT	
PDF1.2	AT5G44420.1-F	GTTCTCTTTGCTGCTTTCGAC	(Santamaría *et al*, 2017)**
	AT5G44420.1-R	GCAAACCCCTGACCATGT	
MYC2	AT1G32640.1-F	TCCGAGTCCGGTTCATTCT	
	AT1G32640.1-R	TCTCGGGAGAAAGTGTTATTGAA	
VSP2	AT5G24770.1-F	ATGCCAAAGGACTTGCCCTA	
	AT5G24770.1-R	CGGGTCGGTCTTCTCTGTTC	
PR1	AT2G14610.1-F	TCAGTGAGACTCGGATGTGC	
	AT2G14610.1-R	CGTTCACATAATTCCCACGA	
UBC	AT5G25760.1-F	GCTCTTATCAAAGGACCTTCGG	
	AT5G25760.1-R	CGAACTTGAGGAGGTTGCAAAG	

* Abu-Romman S, Alzubi J. Transcriptome analysis of Arabidopsis thaliana in response to cement dust. Genes & Genomics. 2016 Sep 1;38(9):865-78.

** Santamaría ME, Martinez M, Arnaiz A, Ortego F, Grbic V, Diaz I. MATI, a novel protein involved in the regulation of herbivore-associated signaling pathways. Frontiers in plant science. 2017 Jun 9;8:975.
