# Supplementary material for: Spider mite egg extract modifies Arabidopsis response to future infestations
Source: Sci Rep. 2021 Sep 6;11:17692. doi: 10.1038/s41598-021-97245-z (PMC8421376; doi:10.1038/s41598-021-97245-z)
Supplement: Supplementary file 5 — Supplementary Information 5. [file 41598_2021_97245_MOESM5_ESM.docx]

**Supplementary information**

Supplemental file 1. DET_List

Supplemental file 2. DEG_List

Supplemental figures. Fig. S1. Representation of the BPs enriched upon mite egg extract treatment. Fig. S2. Representation of the MFs and CCs enriched upon mite egg extract treatment. Fig. S3. Gene Ontology enrichment analysis of the list of DETs. Fig. S4. Representation of the top 50 BPs enriched upon mite egg extract treatment. Fig. S5. Representation of the MFs and CCs enriched upon mite egg extract treatment. Fig. S6. Representation of the relationship of the identified BPs into networks of DEGs. Fig. S7. Representation of the relationships of the identified BPs into networks of DETs. Fig. S8. Main enzymes participating in the inactivation of the JA pathway. Fig. S9. Experimental setup to study *T. urticae* egg extract effect in *A. thaliana*.

Supplementary tables. Table S1 -　Overview of data production quality. Table S2 - Overview of mapping status. Table S3 - Primers used for RT-qPCR validation.
